# Growth in ataxia telangiectasia

**DOI:** 10.1186/s13023-021-01716-5

**Published:** 2021-03-10

**Authors:** Valerie A. I. Natale, Tim J. Cole, Cynthia Rothblum-Oviatt, Jennifer Wright, Thomas O. Crawford, Maureen A. Lefton-Greif, Sharon A. McGrath-Morrow, Haley Schlechter, Howard M. Lederman

**Affiliations:** 1 Forgotten Diseases Research Foundation, Santa Clara, CA USA; 2 UCL Great Ormond Street Institute of Child Health, London, UK; 3 A-T Children’s Project, Coconut Creek, FL USA; 4 Division of Pediatric Allergy and Immunology, The Johns Hopkins Medical Institutions, Baltimore, MD USA; 5 Departments of Pediatrics and Neurology, Johns Hopkins School of Medicine, Baltimore, MD USA; 6 Departments of Pediatrics, Otolaryngology-Head and Neck Surgery, and Physical Medicine and Rehabilitation, Johns Hopkins School of Medicine, Baltimore, MD USA; 7 Children’s Hospital of Philadelphia Division of Pulmonary Medicine and Sleep, Philadelphia, PA USA; 8 Institute for Clinical and Translational Research, Johns Hopkins School of Medicine, Baltimore, MD USA

**Keywords:** Ataxia telangiectasia, Growth charts, Growth, Infections

## Abstract

**Background:**

Ataxia telangiectasia (A-T) is a DNA repair disorder that affects multiple body systems. Neurological problems and immunodeficiency are two important features of this disease. At this time, two main severity groups are defined in A-T: classic (the more severe form) and mild. Poor growth is a common problem in classic A-T. An objective of this study was to develop growth references for classic A-T. Another objective was to compare growth patterns in classic A-T and mild A-T with each other and with the general population, using the CDC growth references. A final objective was to examine the effects of chronic infection on height.

**Results:**

We found that classic A-T patients were smaller overall, and suffered from height and weight faltering that continued throughout childhood and adolescence. When compared to the CDC growth references, the median heights and weights for both male and female patients eventually fell to or below the 3rd centile on the CDC charts. Height faltering was more pronounced in females. Birthweight was lower in the classic A-T group compared to mild A-T and the general population, whereas birth length was not. Finally, we investigated height and BMI faltering in relation to number of infections and found no association.

**Conclusions:**

Classic A-T appears to affect growth in utero. Although children appear to grow well in very early life, faltering begins early, and is unrelenting.

## Introduction

Ataxia telangiectasia (A-T) is a DNA repair disorder characterised by a variety of health problems. They include progressive cerebellar degeneration, immunodeficiency, recurrent infections, radiation sensitivity, and increased risk of cancer, especially of lymphoid origin [1]. Other abnormalities include poor growth, delayed pubertal development and insulin-resistant diabetes [1–5].

A-T is an autosomal recessive condition caused by mutations in the gene *ATM* (ataxia telangiectasia, mutated). *ATM* encodes a large protein kinase (ATM) whose best known role is coordinating the cellular response to DNA double strand breaks. It is also involved in the response to other types of genotoxic stress and, possibly, redox homeostasis [6].

The majority of mutations causing A-T are truncating [7, 8]. In these cases, no ATM protein is detected by Western blotting and no ATM kinase activity is observed. Individuals with these mutations generally have what is called the *classic* clinical presentation of A-T, which is the typical and more severe form of the disorder. Median life expectancy in classic A-T is approximately 25 years [9]. A small number of affected individuals have missense mutations, in-frame mutations or leaky splice-site mutations that allow for the production of residual amounts of functioning ATM protein detectable by Western blots and kinase assays [10]. Patients in this group have been called *atypical*, *variant* or more recently, *mild* [1]. Residual ATM function reduces overall severity of disease, and progression is also slower. An individual in our cohort with the mild form of A-T has survived into the 8th decade; others in the literature have been reported on in their 6th and 7th decades [11, 12].

With the exception of consanguineous populations, A-T affects individuals of all races and ethnicities equally. Incidences as high as 1 in 40,000 and as low as ~ 1 in 300,000 have been reported [13].

Poor growth is a common problem in classic A-T [2, 14–16]. Patients tend to have short stature, and many meet the World Health Organization (WHO) criteria for wasting and stunting (more than 2 standard deviations below mean weight-for-height and mean height for age). Feeding tubes may help, but they are often used after weight loss has become severe, which may limit their utility. Criteria for optimal timing of feeding tube placement are variable. One study advocates placement of tubes in children as young as age 8 to prevent progressive growth failure [2]. Another investigation also endorsed placement at young ages, and before the development of severe nutritional and aspiration induced respiratory compromise [17]. Optimal timing for and the value of feeding tube placement warrant further investigation.

Various factors may influence growth failure in A-T. They include chronic infections [18], IGF-1 hormone deficiency [16, 19], and reduced nutrient intake due to fatigue and swallowing problems [1]. Disease-specific growth references optimise growth assessment in an affected population, and facilitate earlier detection of patterns that are abnormal for a given syndrome. They are particularly useful for a population that tends to plot below the 3rd centile on standard growth charts, as is the case  for the classic A-T population. They have not been made for A-T.

## Materials and methods

### Patients and measurements

All participants met the diagnostic criteria for A-T based on clinical symptoms, as well as laboratory findings of either elevated alpha-fetoprotein (AFP) level, diminished ATM protein, pathogenic *ATM* mutations, and/or increased chromosomal breakage after in vitro exposure to x-rays [20]. They had been seen at the Johns Hopkins Hospital A-T Clinical Center as part of the clinic’s Ataxia Telangiectasia Natural History Study. This study has been running since 1995 and has ongoing Institutional Review Board approval.

### Classification of A-T

We used 8 criteria to classify patients as having mild or classic A-T. They have been adapted from methods previously used [11, 21], and involve neurology, immunology, lung function, AFP level, and mutation, as follows:

- Did the patient have a mutation associated with mild A-T in the literature, or associated with production of some ATM protein by Western blotting?
- Did the patient have relatively mild susceptibility to infections (infection score ≤ 2 on the infection susceptibility scale in Table 1)?
- Could the patient walk at age ≥ 15 years (a score of ≥ 2 on the A-TNEST Walking category [Additional File 1: Table S1]? [22]
- Did the patient have a feeding score ≥ 3 on A-TNEST Self Feeding category at age ≥ 15 years (Additional File 1: Table S2)? [22]
- Was the patient’s forced vital capacity score ≥ 70% of expected value at any age before 30 years?
- Did the patient have a detectable amount of IgA by standard methodology (typically > 7 mg/dL, depending on the assay)?
- Were IgG levels within the normal range for age according to the testing lab providing results, and/or there was no need for IgG replacement therapy to reduce infections?
- Was the patient’s AFP level ≤ 50 ng/ml at age ≥ 7 years? This value was chosen based on our database of AFP values in classic and mild A-T A-T. First, it represents the ~ 10th percentile for 7-year-old classic A-T patients. Because AFP values increase with time [23], 50 was chosen as an outlier in classic A-T. Second, in our mild cohort, AFP values did not tend to increase with time, and the average value for the entire cohort, regardless of age, was 50. Thus, 50 seemed to be an appropriate cutoff value.

Patients earned one point for each yes answer. Scores were calculated as a proportion of yes out of total questions answered, with scores ranging from 0 (lowest score) to 1 (highest possible). Scores ≥ 0.70 were considered to be indicative of mild A-T. The average score for mild A-T was 0.90 (median: 1.00; range 0.70–1.00), and the average for classic A-T was 0.23 (median: 0.21; range 0–0.67).

**Table 1:** Categories for frequency of infections in A-T. Age 10 was used as a cutoff for scoring infection susceptibility because patients become more susceptible to infections as they age and the effects of A-T become progressively more pronounced

Category	Description
1	No increase in susceptibility to infection before age 10
2	Infection frequency greater than unaffected population before age 10, but not significantly impairing (not requiring hospitalization), such as ear infections and sinusitis, or a case of mild pneumonia not requiring hospitalization (as described by a clinician)
3	Frequent and more serious infections before age 10, such as at least one serious pneumonia (e.g. requiring hospitalization), chronic bronchitis, and/or chronic sinusitis refractory to treatment
4	Same as category 3, but serious infections starting before the 2nd birthday

### Inclusion criteria

A diagnosis of A-T was the sole inclusion criterion for the study. Specifically, we included patients regardless of level of immunodeficiency, presence or absence of diabetes or a feeding tube, or degree of neurological progression. This choice was deliberate. Although syndrome-specific growth charts often include only healthy patients with a given syndrome, we did not follow that practice. Immunodeficiency, diabetes, and other chronic problems are integral parts of A-T, and patients were included regardless of their presence or absence so that the composition of study subjects would be representative of the entire A-T population.

### Subjects

When this study began, the clinic had files on 440 confirmed A-T patients, of whom ~ 70% have a genetically confirmed mutation. We used data from 430 classic and mild patients; 10 others were excluded due to absent or unreliable data.

### Anthropometry

Height and weight were measured by trained staff during visits to the A-T Clinical Center, using standardised equipment and protocols. Additional anthropometric data was gathered retrospectively from patient local medical records, which are forwarded to the clinic as part of the A-T Clinical Center’s Natural History study. Each time a patient visits the clinic, we request updated records from paediatricians, family physicians, and specialists.

### Growth data

#### Classic A-T

For classic A-T, we used two sets of growth data. Set 1 consisted of data for 162 patients (81 female, 81 male). This data was extracted from medical records sent to the clinic as part of the Natural History project as well as data from the A-T Clinical Center itself. Data ranged from birth to adulthood. The oldest female patient was age 28.3 years on the date of measurement, and the oldest male was 33.7. The youngest patients were aged < 3 years at the time of visit. We obtained an average of 33 weights and 20 heights per patient in this group, ranging from birth to adulthood. Set 1 is referred to as the *extracted data set*.

Patients in the extracted dataset were chosen so that it would reflect the composition of the overall clinic dataset of 440 patients. Thus, we chose patients from all racial ethnic groups, as well as international and domestic patients.

Table 2 shows the racial and ethnic makeup of the 162 patients in the extracted dataset. Race and ethnicity were unknown in two patients. The patients came from the United States, Australia, Bolivia, Brazil, Canada, Germany, Hong Kong, India, the Netherlands, Turkey, and the UAE.

**Table 2:** Race and ethnicity of study patients. Values do not sum to 100% due mixed race or ethnicity of some individuals

	Classic: extracted dataset	Classic: clinic dataset	All classic	Mild
Number	162	238	400	30
Female (%)	50.0	44.1	46.5	53.3
Male (%)	50.0	55.9	53.5	46.7
Caucasian (%)	76.0	78.7	77.6	100.0
Hispanic (%)	8.4	8.3	8.3	0
African-American (%)	7.2	4.3	5.5	0
Asian, Other (%)	8.4	11.1	10.0	0

Set 2 included height and weight measurements made during visits to the A-T Clinical Center. It did not include anthropometric data extracted from medical records external to the A-T clinic. This data consisted of one to four height and weight measurements from 238 patients (105 female, 133 male; average: 1.3 measurements; median: 1). It is therefore mixed cross-sectional/longitudinal. Data ranged from infancy to adulthood (6 months to 31.8 years for females and 10 months to 34.7 years for males). This set is referred to as the *clinic database set*.

Patients in the clinic database set came from the nations noted above, as well as Angola, Chile, Colombia, Egypt, Greece, Haiti, Italy, Kuwait, Macedonia, Pakistan, Panama, Paraguay, Portugal, Saudi Arabia, Sweden, Trinidad, and the UK.

The total number of classic A-T patients contributing to the growth centile curves was 400,  nearly all the classic patients who had visited the centre. Data from both analysis groups was used to make growth centile curves and the other analyses in this study.

#### Mild A-T

We also extracted longitudinal growth data for 30 mild A-T patients who had visited the clinic (16 female, 14 male). This data was not used for the growth curves, but to examine growth differences between classic and mild A-T. All mild A-T patients were all Caucasian-White and from the United States or Canada (1 patient).

### Birth data

For analysis of birthweight and length, we used data if gestational age was known in weeks and if weight was known in pounds and ounces or to centigrams. Gestational age and precise birthweight were known for 221 classic A-T patients (106 females, 115 males). Birth length was available for 108 patients (52 female, 56 male).

Birth size data was excluded if the mother smoked, used drugs during pregnancy, or had gestational diabetes. A set of triplets was also excluded due to lack of reliable centile data. After exclusions, there were 205 birthweights for classic A-T (101 female, 104 male). No mild A-T patients were excluded from the birth analysis, but gestational age was not known in 3, leaving 27 (15 female, 12 male).

Birthweight centiles were determined using a comprehensive table made from data on for 6.7 million US singleton infants from 22 to 44 weeks completed gestation [24]. Twin births were assessed using Australian data [25]. Birth length was assessed using the Fenton growth charts [26].

### BMI in patients without gastrostomy tubes

A focused analysis compared BMI in classic and mild A-T. This small analysis was a way to examine BMI without the effects of feeding tubes. All patients in the extracted dataset were used; BMI measurements were included before placement of a tube in those who had one. No mild A-T patient received a feeding tube, and all BMI measurements from this group were used.

### Protocol approval and patient consent

All data was gathered as part of the Institutional Review Board-approved Ataxia Telangiectasia Natural History Study at the Johns Hopkins Hospital A-T Clinical Center. Informed consent for collection and use of data is obtained from all patients and/or parents on the occasion of each visit to the A-T Clinical Center.

### Statistical analysis

We used the LMS method to summarise height, weight, and BMI data as growth centile curves. This method creates reference centiles by treating all data as cross-sectional. It estimates the median (M), coefficient of variation (S), and skewness (L) of the data as smooth curves plotted against age, from which selected reference centile curves can be calculated and plotted; data can be converted to internal z-scores [27]. Fitting is done using the *gamlss* package in *R*, where the LMS method corresponds to the BCCG family [28].

Length/height and BMI were also expressed as z-scores and centiles using the US CDC 2000 growth reference.

Statistical significance for continuous variables was calculated using t-tests, with a cutoff of *p* < 0.05 considered significant.

## Results

### Cohort data

#### Sample characteristics

Table 3 shows the number of data points for weight, height and BMI for classic and mild A-T patients.

**Table 3:** Total data points for A-T patients in the extracted dataset

	Classic females	Classic males	All classic A-T	Mild females	Mild males	All mild A-T
Total patients	186	214	400	16	14	30
Weight	2847	2854	5701	330	203	533
Height	1704	1796	3500	249	206	455
BMI	1333	1321	2654	216	137	353

### Growth centile curves for classic A-T

Additional File 2: Figs. S1–S10 are fitted growth curves for height, weight, and BMI. For ease of use, we made two sets of growth charts. For height and weight, they are *Birth—36 months* and *2–18* or *20 years*. For BMI they are ages *2–20 years*. Centile values range from 3 to 97.

Additional File 3: Figs. S11–S16 show individual data points used for construction of the charts.

A freely available calculator is on the Forgotten Diseases Research Foundation’s website [29]. It returns A-T-specific centiles and z-scores for height by age in months or years and months. To facilitate comparison with the unaffected population, data from the CDC centiles are also returned. The calculator is free to use, and the foundation does not see or retain any data entered into it. Calculators for weight and BMI will be added in the future.

### Growth centiles and comparisons to CDC data

**Fig. 1:**
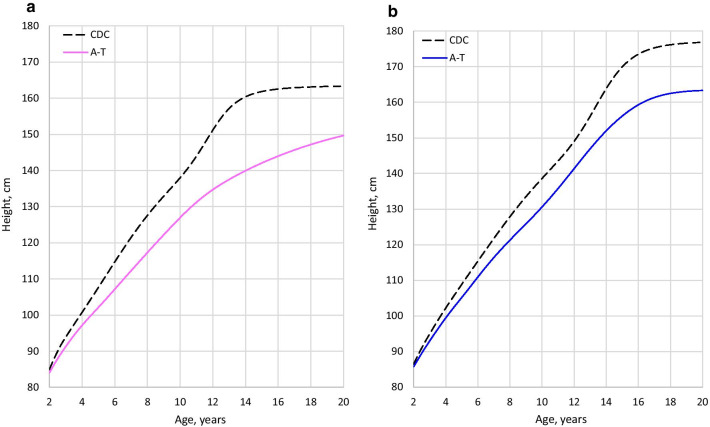
Comparison of height (classic A-T median vs. CDC median). **a** Females. **b** Males

**Fig. 2:**
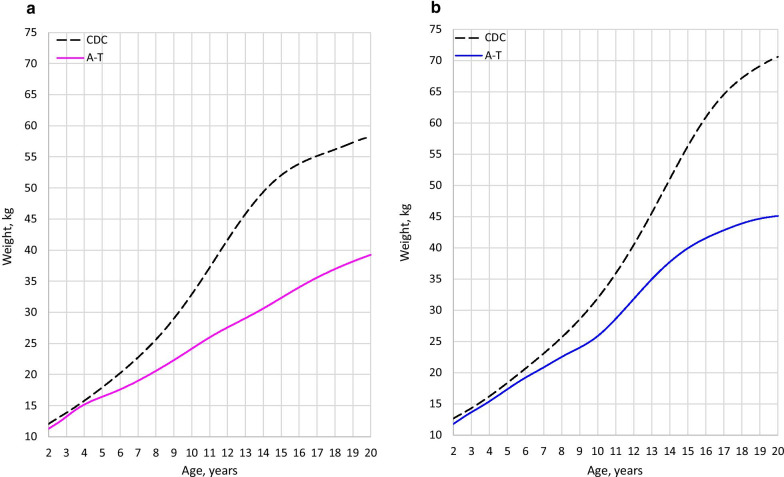
Comparison of weight (classic A-T median vs. CDC median). **a** Females. **b** Males

**Fig. 3:**
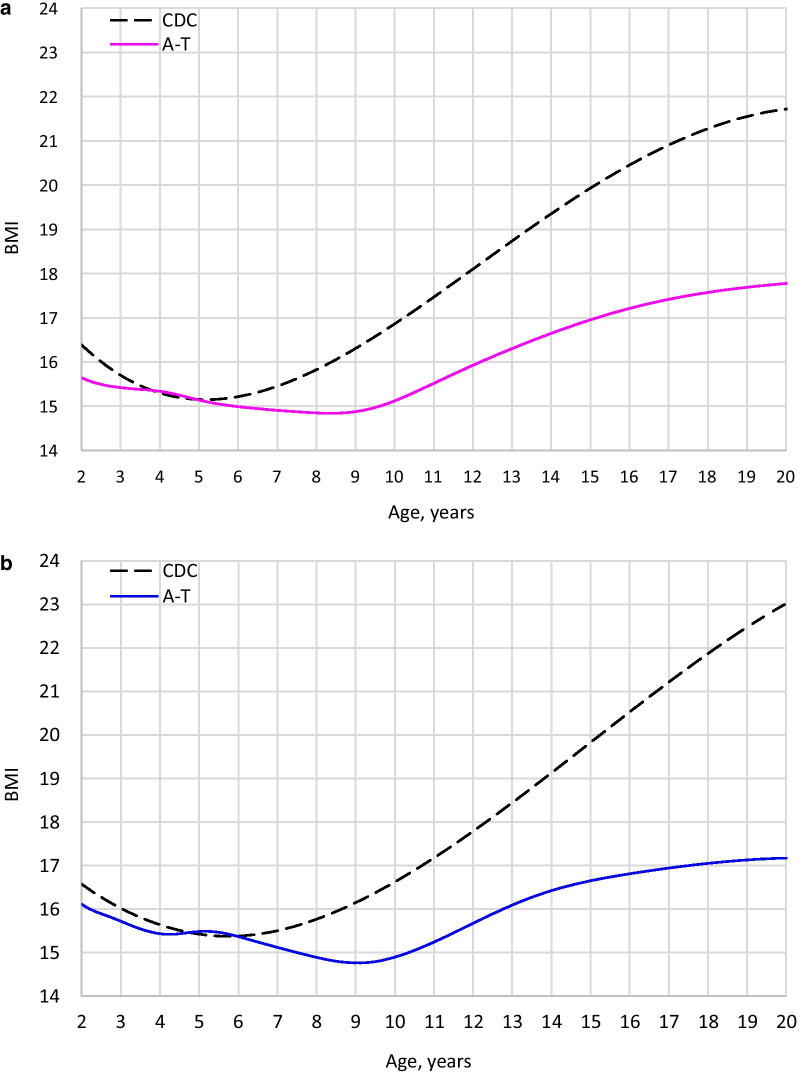
Comparison of BMI (classic A-T median vs. CDC median). **a** Females. **b** Males

Impaired growth was a consistent finding in classic A-T. Although weight, length/height, and BMI tracked with the CDC curves in the toddler years, faltering started early and continued through the teenage years. Figures 1, 2, and 3 show median height, weight, and BMI in A-T compared to the CDC median. In each case, the A-T median falls further below the CDC median with increasing age.

Height faltering started sooner and was more severe in females. Female median height reached the CDC’s 3rd centile at age 6.0 and stayed close to it until crossing below it at age ~11.5. Male height did not fall to the CDC’s 3rd centile until age ~16.5, and did not cross below it (see Additional File 2). Some recovery of centiles occurred in females (Additional File 4: Fig. S17).

Males appeared to have an abbreviated adolescent weight spurt. In females, weight gain with time was nearly linear. Weight faltering occurred in both sexes, started sooner in female patients. The female median crossed below the CDC 3rd at age 10.5, compared to age 14.5 in males (Additional File 2). Female BMI did not fall below the CDC’s 3rd centile, while male BMI went below the 3rd centile around age 16 (Additional File 2). Relatively higher BMI in females may have been related to smaller relative stature.

### Comparisons with mild A-T

#### Birth

Median gestational age in classic A-T was 39 weeks in both sexes. Twenty-two infants were premature (33–36 weeks), and twelve were born at 42 or more weeks. The remaining 171 infants were born between 37 and 41 weeks. Thus, 10.7% were born before term, 83.4% at term, and 5.9% post-term. These figures align well with CDC statistics on pre- and post-term birth rates in the general US population [30].

All 26 mild A-T patients were born at term (averages: 39 weeks in males, 40 weeks in females, and 40 weeks in the entire cohort).

#### Birthweight

The median birthweight centile was 27 in the classic A-T cohort and 61 in the mild cohort (Table 4). A calculation using sample size, mean, and standard deviation indicated that this analysis had 99.99% power to reject the null hypothesis of equal means between the groups.

**Table 4:** Birthweight percentiles in A-T patients, sorted by sex and form of A-T. *Subject numbers were small. See text for details

	Classic females	Mild females	Classic males	Mild males	All classic A-T	All mild A-T
Median %ile	30	53*	26	72*	27	61
Median z-score	− 0.52	0.06*	− 0.64	0.68*	− 0.61	0.55
Number	101	15	104	12	205	27

**Fig. 4:**
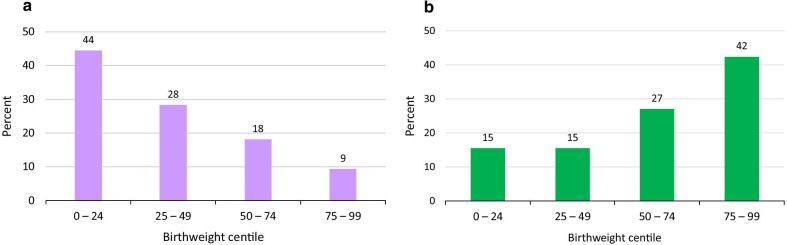
Birthweight percentiles in A-T (using percentiles in the general population as a reference). Data for birthweight came from data sets 1 and 2 (extracted data and clinic database). **a** Classic A-T. **b** Mild A-T. Infants with classic A-T tended to be smaller at birth, while infants with mild A-T tended to be larger

There were striking differences between the mild and classic groups: 72% of classic A-T patients had birthweights below the 50th centile, while 71% of mild A-T patients had birthweights at or above it (Fig. 4).

#### Birth length

**Fig. 5:**
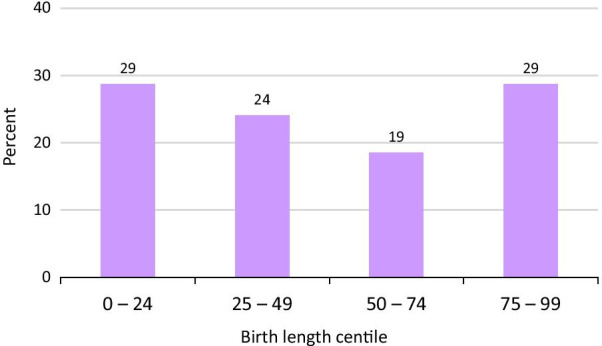
Birth length percentiles in classic A-T. We used percentiles in the general population as a reference [see Methods] [26]. Unlike birthweight, birth length was distributed relatively evenly. There was no sex difference (not shown). We did not have sufficient data for a plot of birth length in mild A-T

Birth length and gestational age in weeks were known for 108 classic A-T patients (52 female, 56 male). Average birth length in both sexes was at the 48th centile based on the Fenton growth charts [26], and spread relatively evenly across the four quartiles (Fig. 5). The figure shows the group as a whole, given that there was no difference between the sexes.

Birth length in mild A-T was known for 6 females and 7 males. This sample size is too small for a meaningful analysis, but mean length for the group was at the 62nd centile.

### Postnatal growth

We examined height and BMI from after birth until adulthood. Weight for age was not included in this analysis; given the generally short stature of classic A-T patients, we believed that BMI was a better indicator of nutritional status.

### Height

**Fig. 6:**
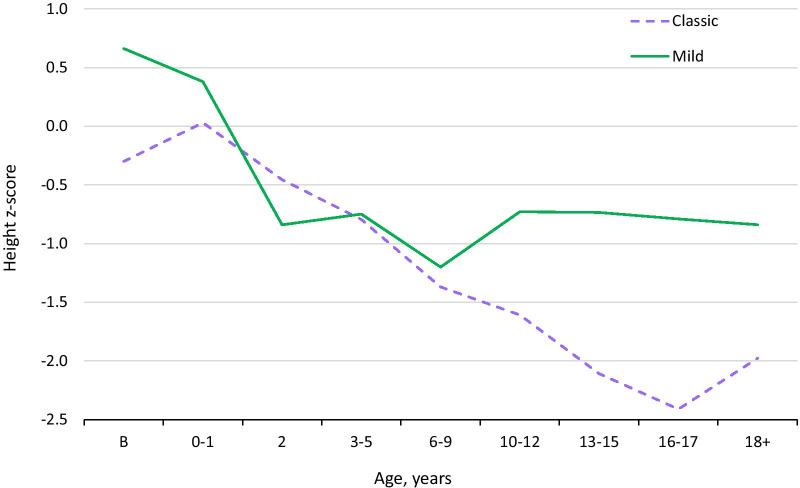
Median height z-score by age in mild and classic A-T, sexes combined (z-scores with respect to CDC values)

Figure 6 compares median height in classic and mild A-T patients with the CDC median. Faltering was evident in the classic cohort, but was less pronounced in mild A-T. By adulthood, 88% of women and 62% of men with classic A-T had heights below the CDC’s 10th centile, and only 1 had a height above the 50th. In contrast, in mild A-T, 3 adult men and 1 woman had heights above the CDC's 80th centile, with one man having a height above the 99th. Importantly, the centile drop in mild A-T may have been due to lack of childhood and adolescent growth data for some of the mild A-T patients, including data for the tallest patients.

The WHO defines stunting as a height at least two standard deviations below the mean. Stunting was common in classic A-T. In classic A-T patients age 13 or older, it occurred in 67% of females and 41% of males (it was therefore 1.5 times more common in females; Additional File 4: Fig. S18). Of 26 mild A-T patients over age 13, only one was stunted.

A sex-specific height difference appears to exist in mild A-T as well as in classic A-T. Additional File 4: Fig. S19 shows that at ages 10 and above, male heights were close to the CDC average, while female heights were well below it. In spite of this fact, females with mild A-T were considerably taller than those with classic A-T. Additional File 4: Fig. S20 shows median height in females with mild A-T as a function of age. Faltering occurred before age 2, but appeared to level off after that time. We were not able to make a similar figure for males, due to sparse data.

### Height and mortality in classic A-T

**Fig. 7:**
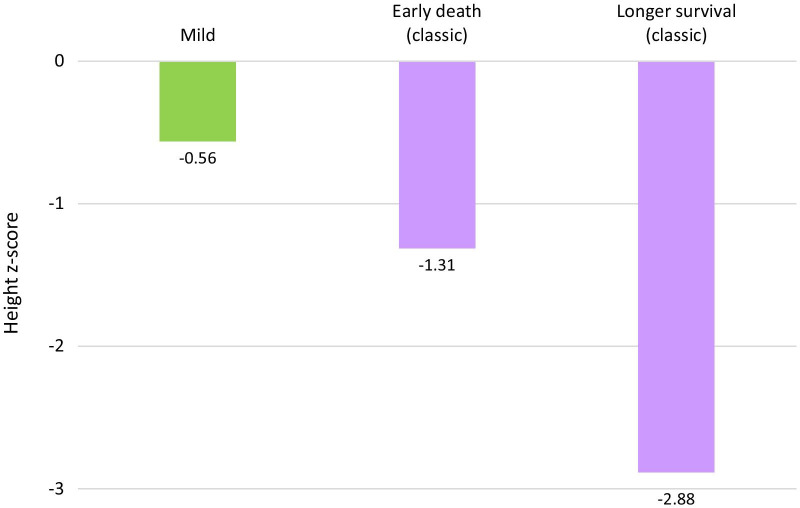
Median height z-score at age 10–14 in mild and classic A-T, split by length of survival (compared to CDC data). This age range was chosen to facilitate comparison between the oldest patients in the early death cohort and other patients. Mild: mild A-T (41 data points/19 patients). Longer survival: patients surviving past age 25 (99 data points/35 patients). Early death: patients dying by age 15.0 (16 data points/9 patients)

To explore the link between growth and early mortality, we subdivided classic A-T patients into two groups: an early death cohort (died by age 15.0) and a longer-surviving cohort (survived past age 25.0). We compared CDC height z-scores for age 10–14 in these two groups and in mild A-T patients. Figure 7 shows that patients who died young were shortest, with mild A-T patients being the tallest.

### Effect of infection on height

We used medical records to classify the 162 extracted data set patients into 4 groups based on frequency and severity of infections before their 10th birthdays See *Methods* and Table 1 for criteria. Briefly, patients in groups 1 and 2 were designated as being *less susceptible* to infection, and patients in groups 3 and 4 were designated *more susceptible*. We classified 144 patients using this system (17 were too young and data was insufficient data for 1 other).

We compared height in older patients aged  ≥ 13 years (88 total patients). The median height z-score was the same in both groups, at − 2.08 SD. The mean in the less susceptible group was − 2.07 SD, and − 2.17 in the more susceptible group (difference not statistically significant).

In addition, 55% of stunted patients had high susceptibility to infection and 45% had low susceptibility. These proportions were essentially the same among patients were not stunted: 54% had high susceptibility to infection and 46% had low susceptibility.

### BMI in patients without gastrostomy tubes

**Fig. 8:**
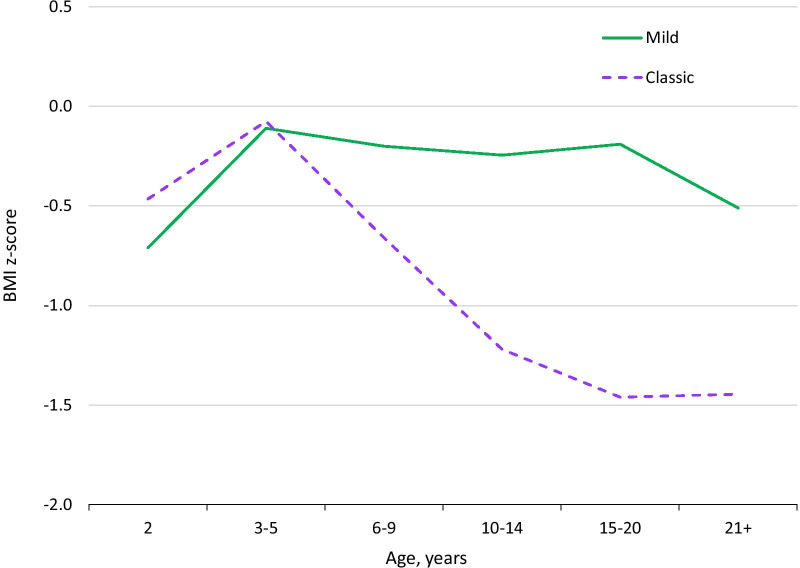
BMI in classic and mild A-T. BMI z-score by age in A-T. BMI in classic A-T falls with age. Mild A-T shows no age trend. The figure uses data from the 162 patients in the extracted dataset and 31 patients with mild A-T

We examined BMI in patients without gastrostomy tubes as a way of analysing the effects of A-T in the absence of surgical interventions aimed at improving nutrient intake. Median BMI peaked between the 3rd and 6th birthdays and steadily declined thereafter (Fig. 8, dashed violet line). Faltering did not occur in mild A-T (solid green line). In classic A-T, there was no significant difference between males and females until later ages, when male BMI recovered slightly (Additional File 4: Fig. S21). When we examined BMI in classic A-T patients with and without feeding tubes, the differences were not statistically significant.

**Fig. 9:**
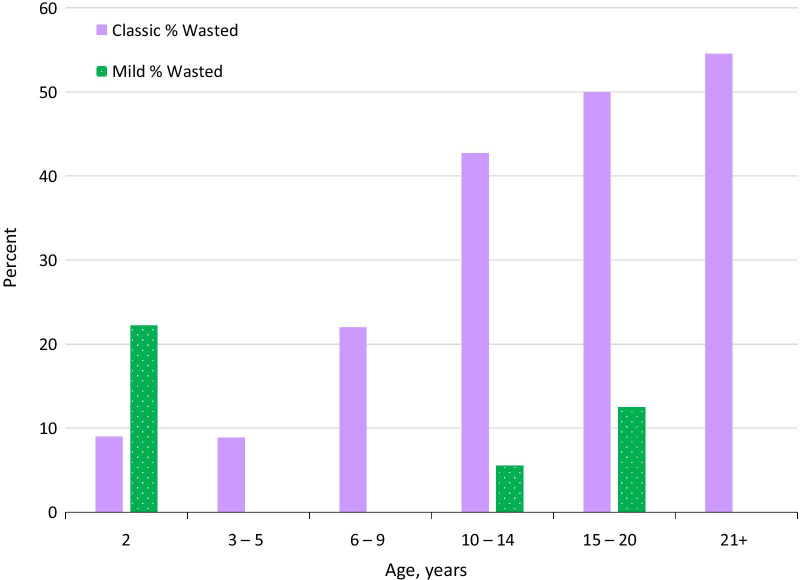
Prevalence of wasting (BMI z-score ≤ –2) by age in A-T patients. Solid violet bars: classic A-T. Dotted green bars: mild A-T

As classic A-T patients aged, an increasing proportion met or exceeded the WHO definition of moderate malnutrition, which is a BMI of 2 or more SDs below the mean (Fig. 9, solid violet bars). The pattern was different in mild A-T (dotted green bars).

BMI in many classic A-T patients was far below the − 2 SD cutoff, particularly in adolescence. We had BMI measurements for 78 patients aged between 11 and 20. Twenty-two of them had BMI below − 3 SDs (28.2%; WHO-defined severe malnutrition).

We also looked at minimum and maximum BMI centiles in classic A-T. The minimum values were always low, and fell with time to – 8 SD by age 17 (Table 5). Maximum values hovered around 2 until age 15, and then declined thereafter to – 0.03 SD by the 20s. This pattern was not observed in mild A-T patients (green line in Fig. 6).

**Table 5:** Highest and lowest BMI values in classic A-T patients. The value of 1.93 was in a 15-year-old male. This same patient had the BMI z-score of -0.03 at age 24 (21+ age group). The next highest BMI in a 20-year-old patient was -1.18 SDs

Age group (years)	Lowest BMI z-score	Highest BMI z-score
2	− 3.23	2.15
3–5	− 3.74	2.16
6–9	− 4.36	2.06
10–14	− 7.58	2.01
15–20	− 8.62	1.93
21+	− 8.48	− 0.03

Some classic A-T patients were overweight or mildly obese. For example, from ages 11–20, 6 patients had BMIs between the 86th and 98th centiles, meeting the definition for overweight or obesity. We had data for three of these patients after age 21; their highest BMIs after that age had declined to the 71st, 49th, and 45th centiles. Overweight in classic A-T occurred most frequently at ages 3–5 (17% of children in this age group), and did not occur in adults. No mild A-T were obese, 5 were overweight before age 15, and none over 15 were overweight.

## Discussion

This study analysed growth in a large and diverse cohort of patients with classic and mild A-T. Overall, we found a pattern of growth faltering in classic A-T that did not occur or was less severe in people with mild A-T.

As noted in the Inclusion criteria, a diagnosis of A-T was the sole criterion for inclusion in the study. This choice was deliberate so that our growth references would reflect the entire A-T population. Immunodeficiency, diabetes, malnutrition, and other chronic problems are integral parts of A-T. As a result, a large majority of A-T patients use daily interventions including antibiotics, steroids, supplemental immune globulins, and/or feeding tubes. If we had excluded patients with one or more of these conditions or interventions, the growth references would not reflect the population as a whole.

Patients with classic A-T grew well up to age 2. After then, height, weight, and BMI faltered continuously, and growth parameters eventually fell to or below the CDC's 3rd centile. Females fell further below the CDC median height than males, and did so at an earlier age. However, there was recovery of height centiles in females after age 15. This effect may have been due to lifelong greater height  in patients who survived longer, but further studies would be required to make a determination. However, our finding that classic A-T patients who survived after age 25.0 were larger overall than those who died by age 15.0 lends support to the idea.

Interestingly, BMI faltering occurred earlier than might have been predicted by the onset of swallowing difficulties, which tend to begin early in the second decade of life [31]. This fact suggests that other effects of A-T may be responsible for height and weight faltering. These effects may include poor appetite, which is a trait commonly cited by caregivers, and physiological factors that are currently not known or understood.

Approximately 14.5% of our BMI data in classic A-T came from patients with tubes. When we compared BMI in patients with and without tubes, we found that BMI did not differ significantly. The reasons for this difference are unknown, but may be related to postponement of tube use until a patient’s nutritional status has become severe, use of tubes in patients with nutritional problems that are more severe than those who do not have them, and the possibility that the tubes were present but not used.

Chronic infection—especially gut infection—in children living in developing nations has a documented effect on growth [32–34]. However, the association between infection and growth failure is complex and still not fully understood [35]. A review of Turkish patients noted that growth retardation was more frequent in patients with chronic sinopulmonary infections [18]. Given that many patients with A-T suffer from chronic respiratory tract infections, we examined the relationship between infection and linear growth. We did not find correlations between growth and frequency of infections in A-T. Furthermore, in many patients, growth faltering occurred before the onset of chronic infections.

Overall, the pattern of growth faltering seen here agrees with earlier findings by Nissenkorn et al., who noted that poor growth is a part of A-T and is more severe in females [3]. However, unlike that study, we found that degree of growth faltering correlated with age at death. Given that infections alone did not correlate with smaller size, there is an implication that factors beyond chronic infections contribute to growth failure and survival.

Birthweight in classic A-T patients was relatively low in this study, while birth length was similar to that for the unaffected population. Of note, *atm* null mice, a murine model of A-T, are smaller at birth and weigh less than unaffected or *atm* ± mice [36, 37]. Indeed, in one study, ATM knockout mice of different strains weighed on average, 72–87% as much as *atm*+/+ mice of the same sex and strain at five weeks of age [38].

Height faltering in classic A-T occurs in essentially every patient with classic A-T and is an unrelenting problem. This faltering appears to worsen in adolescence—a period when a growth spurt occurs in the unaffected population.

### Importance of growth charts

The heights and weights of many classic A-T patients are more than 2 standard deviations below the mean of the US CDC growth curves. Because data plotted below the bottom line on a growth chart can be difficult to interpret, syndrome-specific growth charts allow more precise assessment of growth of individual patients. A comparison with CDC or other local growth charts facilitates further analysis.

### Strengths and limitations of the study

A major strength of this study is that it used a large, genetically and ethnically diverse cohort of patients seen at the A-T Clinical Center at Johns Hopkins Hospital. Our growth charts were based on ~ 5700 data points for weight and ~ 3500 data points for height from 400 patients, which is also a significant strength. Such large patient numbers are not easily obtained in studies of diseases as rare as A-T. In addition, the detailed nature of the records gathered by the clinic has facilitated not only a large amount of anthropometric data, but also data for other factors that may contribute to poor growth, including prevalence of infections, environment, early death, and other factors.

One limitation of the study is that longitudinal data can be limited if patients do not return for follow-up visits. It can also be limited because the clinic does not always receive a full set of records for every patient. There also may be an acquisition bias, because at all ages sicker individuals and those with fewer economic resources may be less likely to travel out-of-state or out-of-country to our clinic. This last limitation was mitigated to some extent because some parents sent medical record updates in the absence of clinic visits. Full sets of records can be difficult to obtain for mildly affected patients diagnosed as teenagers or adults, or for patients whose families move to a new state or country. In addition, no standardised set of most important laboratory tests has established for monitoring A-T.

### Future directions

A study of the influence of feeding tubes and other interventions on overall outcomes in A-T would be a valuable addition to the literature on A-T. It will be important to determine if tube feeding tubes improve life expectancy, resistance to infection, lung function, fatigue levels, pubertal outcomes (particularly in females) and other problems in A-T.

A-T-specific growth charts will be useful in clinical trials in A-T. For example, an effective intervention given at a young age may affect growth, and these changes will be more easily seen on a growth chart specific to A-T. Furthermore, comparing the effects of variables known to influence growth could also provide insight into the interplay between nutrition, growth, and health in A-T patients. It remains to be seen if dietary or hormonal interventions to improve growth will have effects on other aspects of A-T.

## Conclusions

Classic A-T appears to affect growth in utero. Although children appear to grow well in very early life, faltering begins early and is unrelenting. Growth is less affected in mild A-T. The growth charts presented here should be of use to clinicians, researchers, parents, and anyone else wanting to monitor growth in A-T.

## Supplementary information

**Additional file 1.** Tables S1 and S2 (A-TNest scores).

**Additional file 2.** Growth charts for A-T.

**Additional file 3.** Dot plots showing raw data for growth charts.

**Additional file 4.** Additional figures S17–S21.

**Additional file 5.** LMS data.

## Data Availability

The datasets supporting the conclusions of this article are included within the article and its additional files. Additional file 3 shows raw data as dot plots on centile curves. Additional file 5 has LMS data for height, weight, and BMI in A-T patients. CDC centile data is publicly available.

## Abbreviations

A-T: Ataxia telangiectasia
ATM: Ataxia telangiectasia, mutated
CDC: Centers for Disease Control
WHO: World Health Organization
