# Correction to: Growth in ataxia telangiectasia

**DOI:** 10.1186/s13023-021-01891-5

**Published:** 2021-06-01

**Authors:** Valerie A. I. Natale, Tim J. Cole, Cynthia Rothblum-Oviatt, Jennifer Wright, Thomas O. Crawford, Maureen A. Lefton-Greif, Sharon A. McGrath-Morrow, Haley Schlechter, Howard M. Lederman

**Affiliations:** 1 Forgotten Diseases Research Foundation, Santa Clara, CA USA; 2 UCL Great Ormond Street Institute of Child Health, London, UK; 3 A-T Children’s Project, Coconut Creek, FL USA; 4 Division of Pediatric Allergy and Immunology, The Johns Hopkins Medical Institutions, Baltimore, MD USA; 5 Departments of Pediatrics and Neurology, Johns Hopkins School of Medicine, Baltimore, MD USA; 6 Departments of Pediatrics, Otolaryngology-Head and Neck Surgery, and Physical Medicine and Rehabilitation, Johns Hopkins School of Medicine, Baltimore, MD USA; 7 Children’s Hospital of Philadelphia Division of Pulmonary Medicine and Sleep, Philadelphia, PA USA; 8 Institute for Clinical and Transla- Tional Research, Johns Hopkins School of Medicine, Baltimore, MD USA

## Correction to: Orphanet J Rare Dis (2021) 16:123 10.1186/s13023-021-01716-5

Following the publication of the original article [1] the authors became aware of an error in Fig. 7: The labels over the purple bars were unfortunately interchanged.

**Fig. 7:**
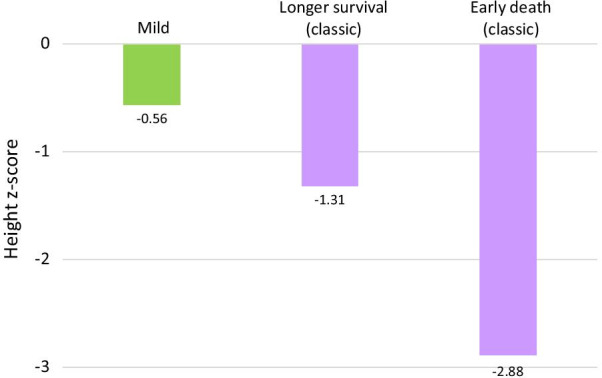
Median height z-score at age 10–14 in mild and classic A-T, split by length of survival (compared to CDC data). This age range was chosen to facilitate comparison between the oldest patients in the early death cohort and other patients. Mild: mild A-T (41 data points/19 patients). Longer survival: patients surviving past age 25 (99 data points/35 patients). Early death: patients dying by age 15.0 (16 data points/9 patients)

− 1.31 corresponds to ‘Longer survival (classic)’, while − 2.88 corresponds to ‘Early death (classic)’.

The correct Fig. 7 is included in this Correction and has already been updated in the original article.
