# Supplementary material for: Growth in ataxia telangiectasia
Source: Orphanet J Rare Dis. 2021 Mar 10;16:123. doi: 10.1186/s13023-021-01716-5 (PMC7945359; doi:10.1186/s13023-021-01716-5)
Supplement: Supplementary file 1 — Additional file 1. Tables S1 and S2 (A-TNest scores). [file 13023_2021_1716_MOESM1_ESM.pdf]

Additional file 1

Tables S1 and S2

Category	Description
0	Requires vertical support
1	Massive lateral support (above elbow or waist held)
2	Some lateral support (below elbow or hand held, elbow relaxed)
3	Only touch support needed
4	No support, wide base or takes corrective stagger steps
5	No support, normal speed with mild path deviations or corrective steps
6	Normal path width without corrective steps

Table S1. A-TNEST categories for walking.

Category	Description
0	No functional self-feeding
1	Needs help with some feeding, even with finger feeding
2	Needs help with some feeding, even with all utensils
3	Needs help with some feeding, even with spoon
4	Independent feeding only with adaptations
5	Normal for age

Table S2. A-TNEST categories for self-feeding.
