# Supplementary material for: Growth in ataxia telangiectasia
Source: Orphanet J Rare Dis. 2021 Mar 10;16:123. doi: 10.1186/s13023-021-01716-5 (PMC7945359; doi:10.1186/s13023-021-01716-5)
Supplement: Supplementary file 2 — Additional file 2. Growth charts for A-T. [file 13023_2021_1716_MOESM2_ESM.pdf]

Additional file 2

Growth charts for classic ataxia telangiectasia

Additional figures S1 – S10

This file contains growth charts for height, weight, and BMI. They have been designed for clinical use.

The Forgotten Diseases Research Foundation has created a free online calculator for A-T. It has data from birth to adulthood in monthly increments. Users enter an age and a value such as height. The calculator returns syndrome-specific centiles and z-scores, as well as centiles and z-scores from the CDC data. This design therefore facilitates analysis of growth according to two references.

The calculator may be accessed at www.forgottendiseases.org under the Calculators tab. The Forgotten Diseases Research Foundation does not save or view any data entered into the calculator.

Individual growth charts are also available for download at www.forgottendiseases.org under the Rare Diseases tab.

Name: ___________________________

Green line shows CDC 3rd percentile

Date of birth: _____/______/_________

Girls with Classic A-T: Weight, Birth – 36 months

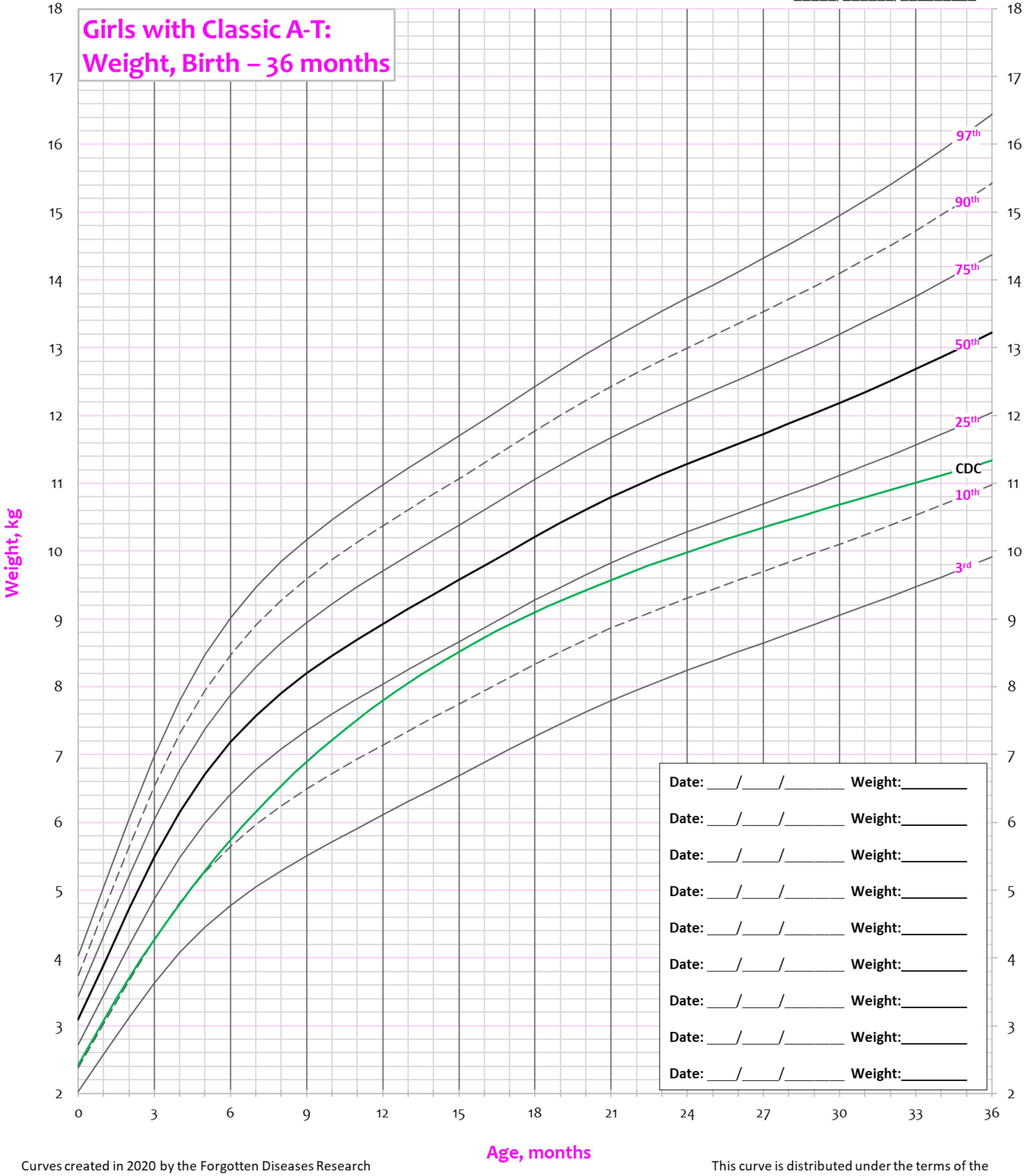

Date: ____/_____/________ Weight:_________

Date: ____/_____/________ Weight:_________

Date: ____/_____/________ Weight:_________

Date: ____/_____/________ Weight:_________

Date: ____/_____/________ Weight:_________

Date: ____/_____/________ Weight:_________

Date: ____/_____/________ Weight:_________

Date: ____/_____/________ Weight:_________

Date: ____/_____/________ Weight:_________

Curves created in 2020 by the Forgotten Diseases Research Foundation and Professor Tim Cole (University College London). Data source: A-T Clinical Center at Johns Hopkins Hospital.

This curve is distributed under the terms of the Creative Commons Attribution 4.0 International License (http://creativecommons.org/licenses/by/4.0/)

Name: ___________________________

Green line shows CDC 3rd percentile

Date of birth: _____/______/_________

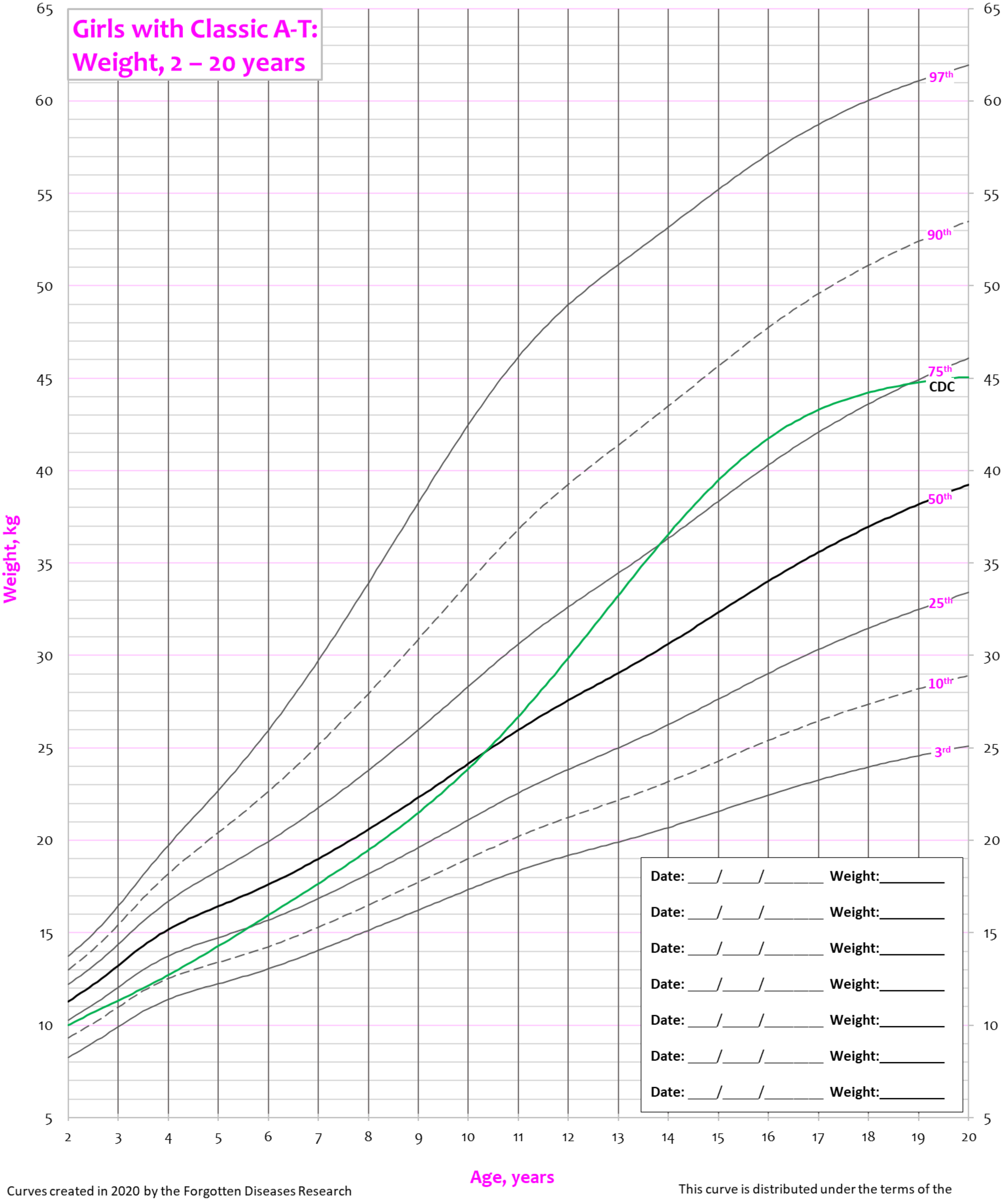

Curves created in 2020 by the Forgotten Diseases Research Foundation and Professor Tim Cole (University College London). Data source: A-T Clinical Center at Johns Hopkins Hospital.

This curve is distributed under the terms of the Creative Commons Attribution 4.0 International License (http://creativecommons.org/licenses/by/4.0/)

Name: ____________________________

Date of birth: _____/______/_________

Green line shows CDC 3rd percentile

Girls with Classic A-T: Height, Birth – 36 months

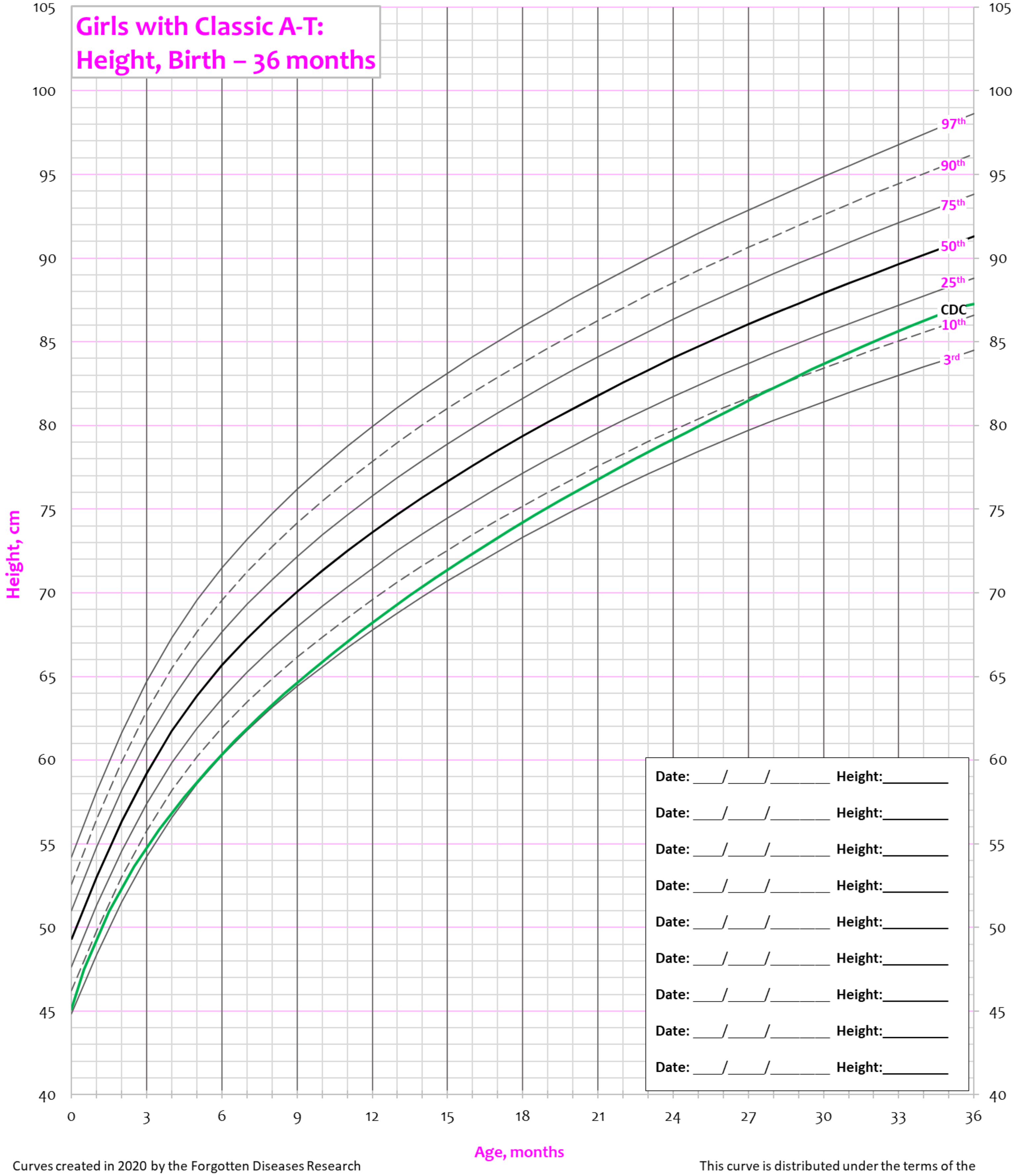

Date: ____/_____/________	Height:_________
Date: ____/_____/________	Height:_________
Date: ____/_____/________	Height:_________
Date: ____/_____/________	Height:_________
Date: ____/_____/________	Height:_________
Date: ____/_____/________	Height:_________
Date: ____/_____/________	Height:_________
Date: ____/_____/________	Height:_________
Date: ____/_____/________	Height:_________

Curves created in 2020 by the Forgotten Diseases Research Foundation and Professor Tim Cole (University College London).
Data source: A-T Clinical Center at Johns Hopkins Hospital.

This curve is distributed under the terms of the Creative Commons Attribution 4.0 International License (http://creativecommons.org/licenses/by/4.0/)

Name: ___________________________

Date of birth: _____/______/_________

Green line shows CDC 3rd percentile

Girls with Classic A-T: Height, 2 – 18 years

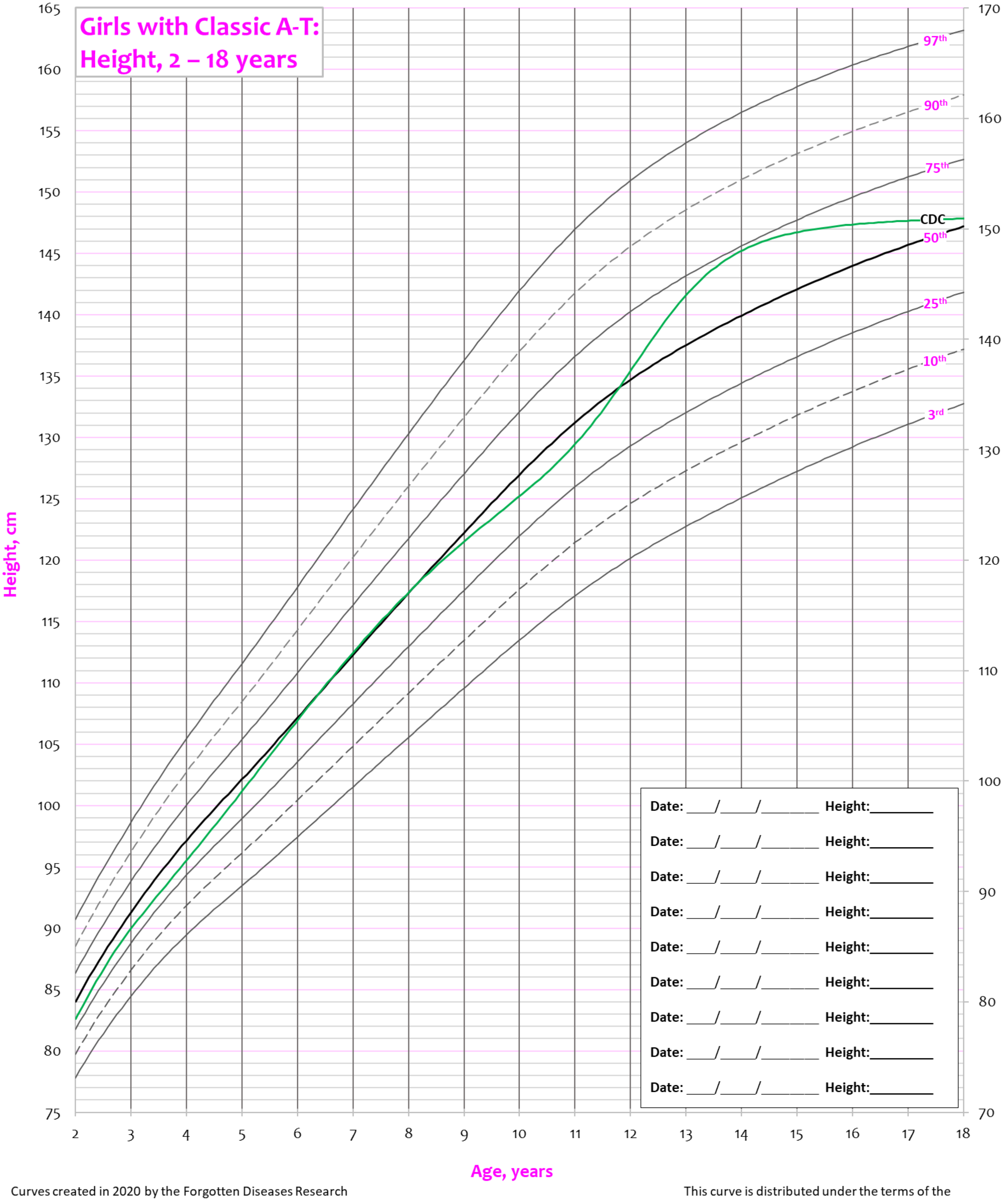

Curves created in 2020 by the Forgotten Diseases Research Foundation and Professor Tim Cole (University College London). Data source: A-T Clinical Center at Johns Hopkins Hospital.

This curve is distributed under the terms of the Creative Commons Attribution 4.0 International License (http://creativecommons.org/licenses/by/4.0/)

Name: ____________________________

Date of birth: _____/______/_________

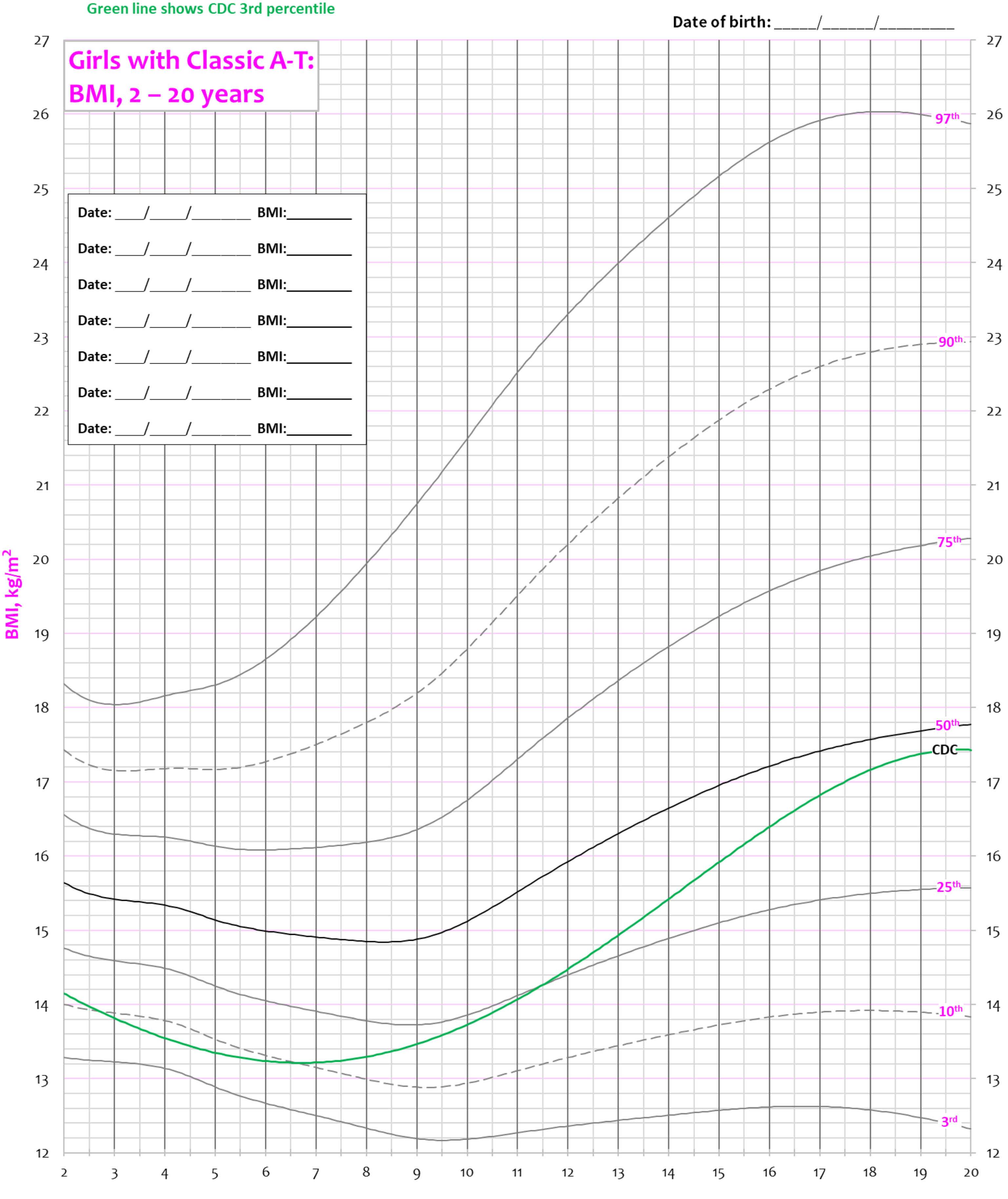

Curves created in 2020 by the Forgotten Diseases Research Foundation and Professor Tim Cole (University College London). Data source: A-T Clinical Center at Johns Hopkins Hospital.

This curve is distributed under the terms of the Creative Commons Attribution 4.0 International License (http://creativecommons.org/licenses/by/4.0/)

Name: ______________________________

Red line shows CDC 3rd percentile

Date of birth: _____/______/_________

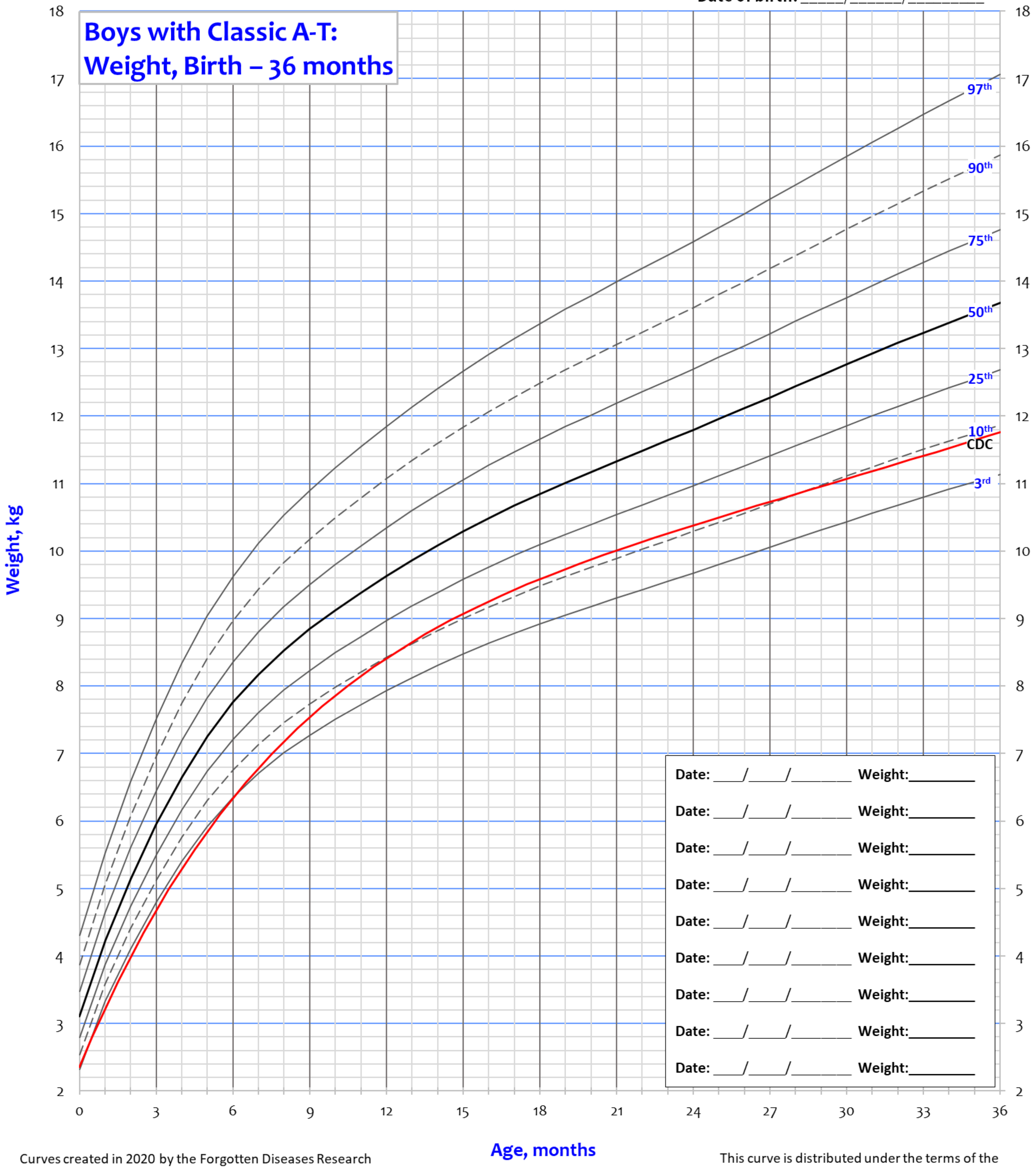

Curves created in 2020 by the Forgotten Diseases Research Foundation and Professor Tim Cole (University College London). Data source: A-T Clinical Center at Johns Hopkins Hospital.

This curve is distributed under the terms of the Creative Commons Attribution 4.0 International License (http://creativecommons.org/licenses/by/4.0/)

Name: ____________________________

Red line shows CDC 3rd percentile

Date of birth: _____/______/_________

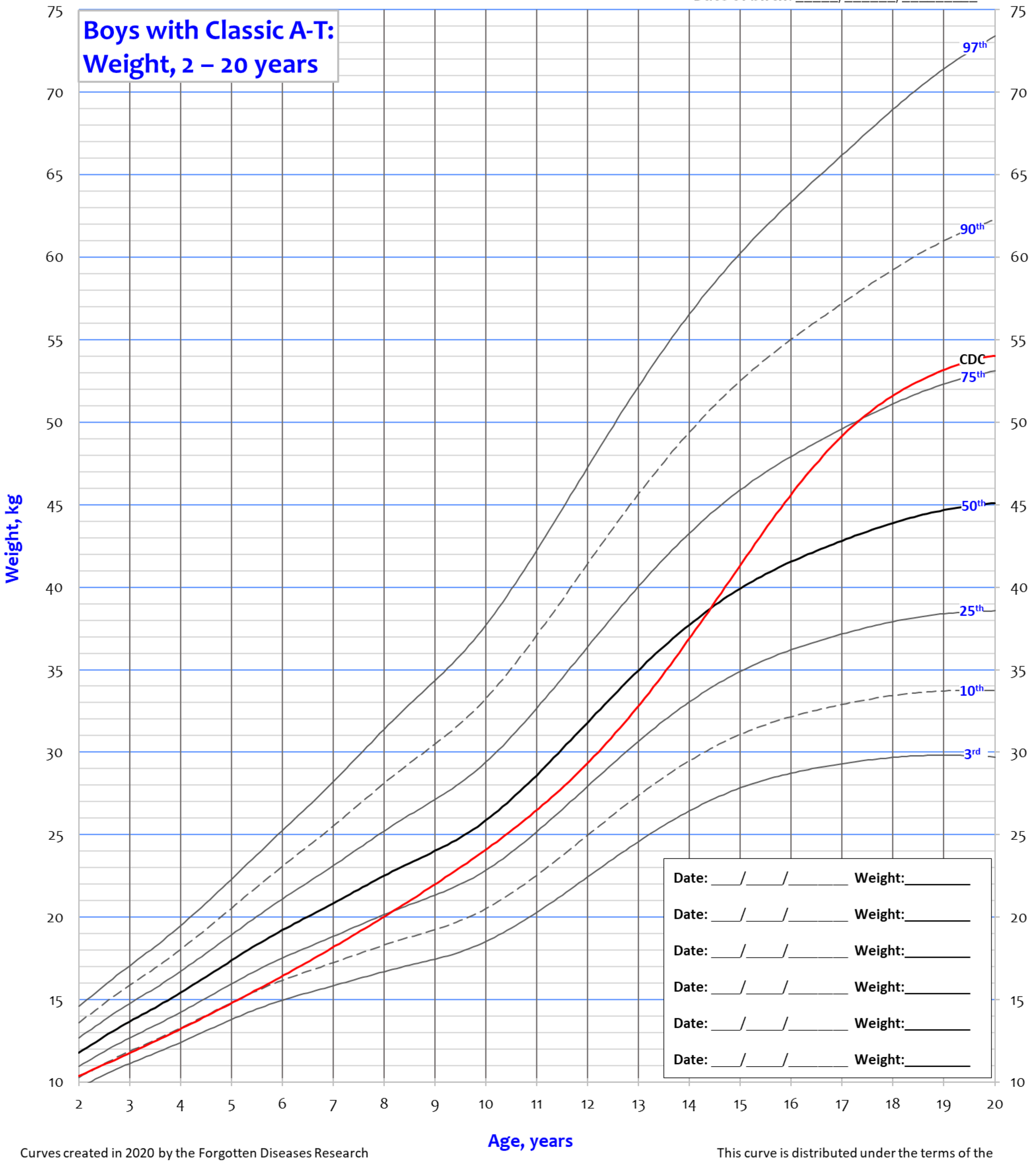

Curves created in 2020 by the Forgotten Diseases Research Foundation and Professor Tim Cole (University College London).
Data source: A-T Clinical Center at Johns Hopkins Hospital.

This curve is distributed under the terms of the Creative Commons Attribution 4.0 International License (http://creativecommons.org/licenses/by/4.0/)

Name: ____________________________

Date of birth: _____/______/_________

Red line shows CDC 3rd percentile

Boys with Classic A-T: Height, Birth – 36 months

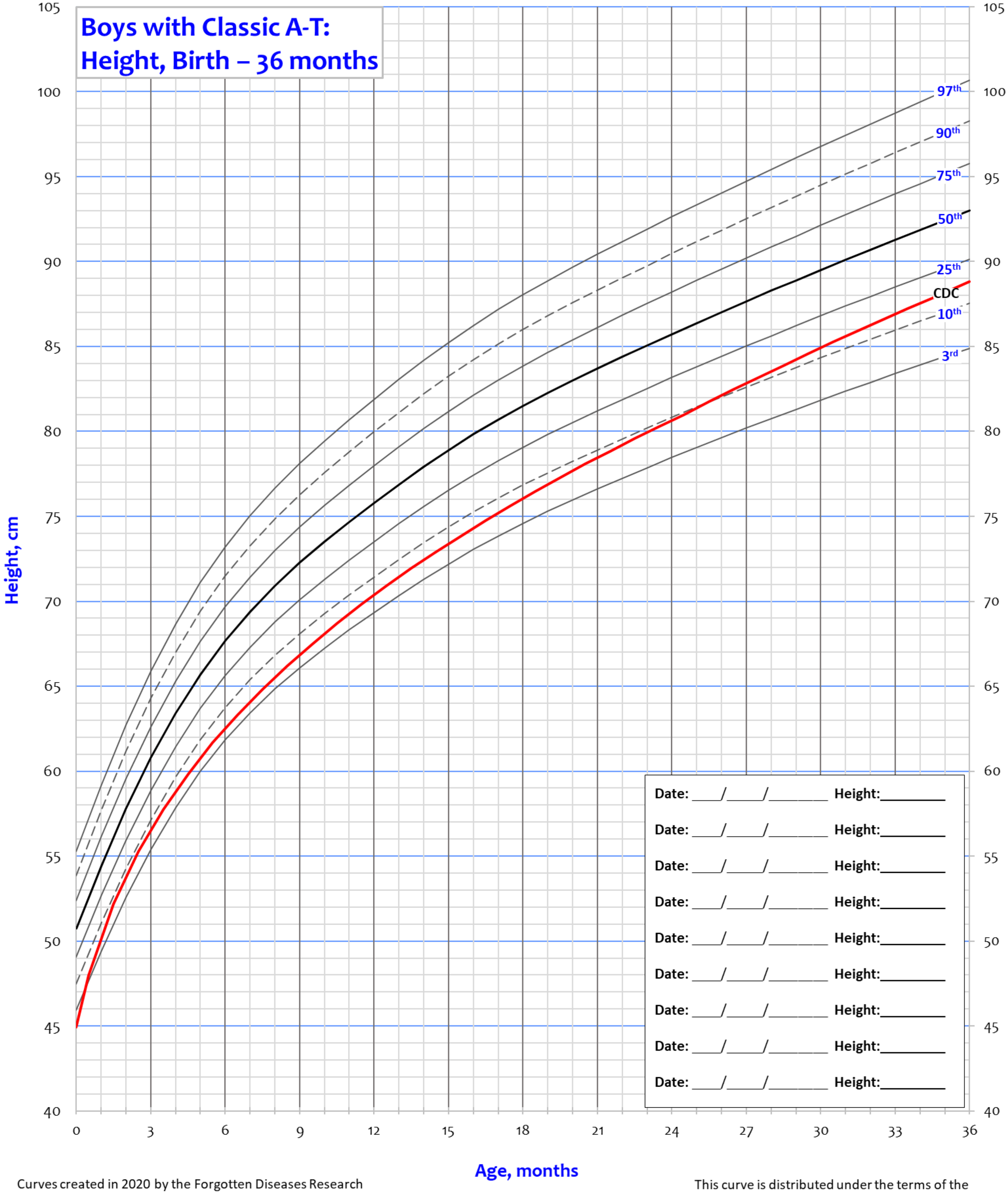

Curves created in 2020 by the Forgotten Diseases Research Foundation and Professor Tim Cole (University College London). Data source: A-T Clinical Center at Johns Hopkins Hospital.

This curve is distributed under the terms of the Creative Commons Attribution 4.0 International License (http://creativecommons.org/licenses/by/4.0/)

Name: ___________________________

Red line shows CDC 3rd percentile

Date of birth: _____/______/_________

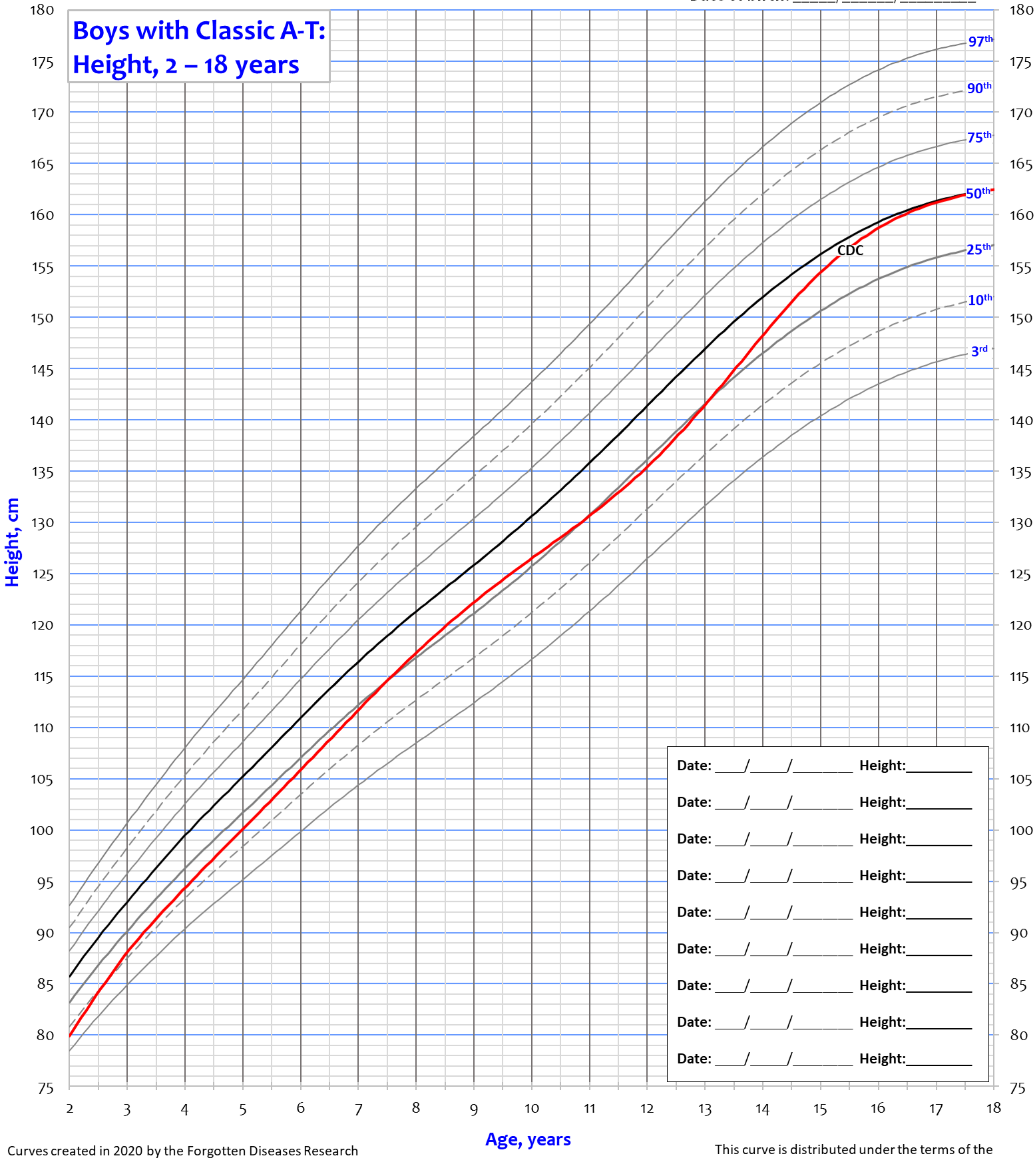

Curves created in 2020 by the Forgotten Diseases Research Foundation and Professor Tim Cole (University College London).
Data source: A-T Clinical Center at Johns Hopkins Hospital.

This curve is distributed under the terms of the Creative Commons Attribution 4.0 International License (http://creativecommons.org/licenses/by/4.0/)

Name: ____________________________

Red line shows CDC 3rd percentile

Date of birth: _____/______/_________

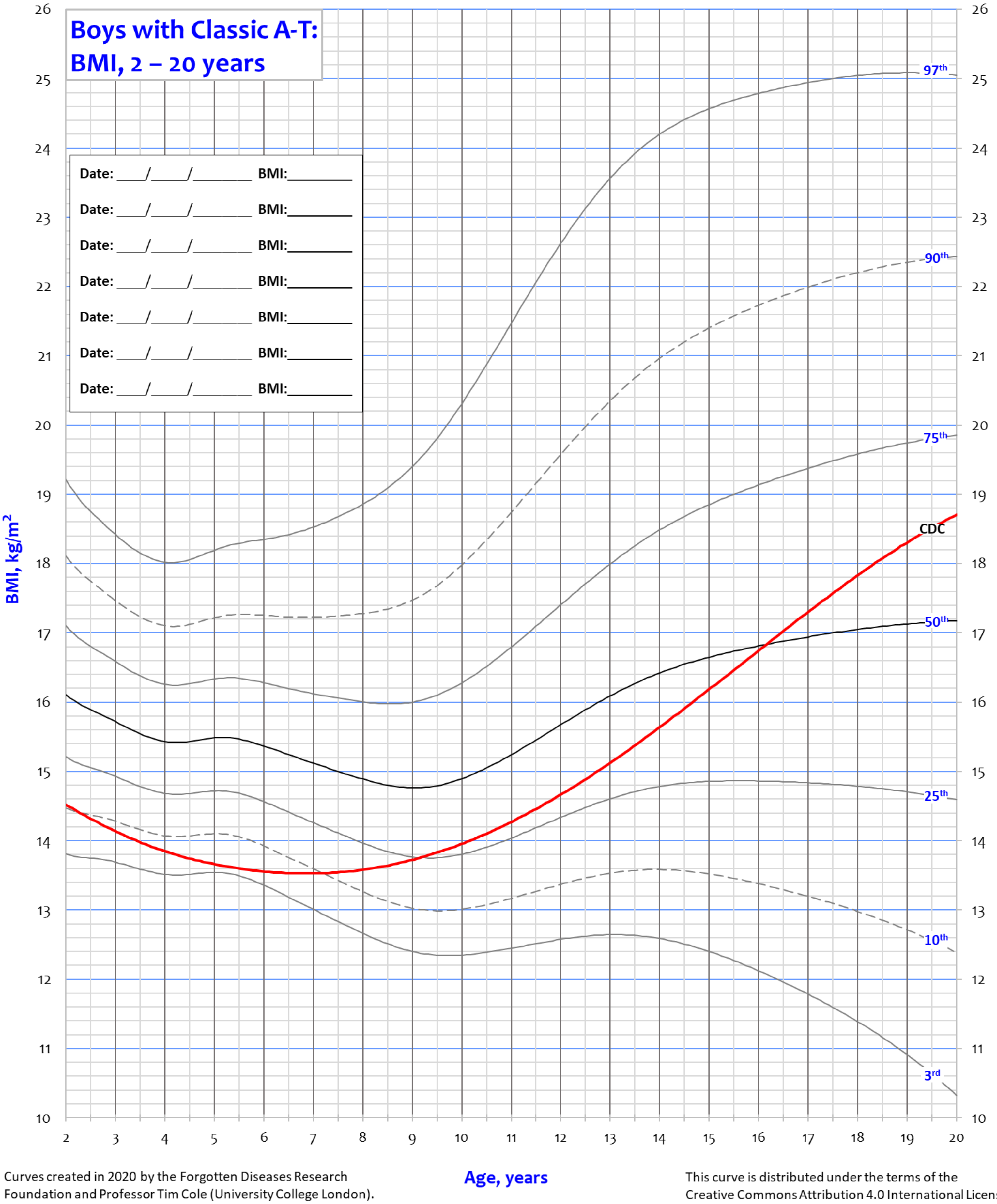

Curves created in 2020 by the Forgotten Diseases Research Foundation and Professor Tim Cole (University College London). Data source: A-T Clinical Center at Johns Hopkins Hospital.

This curve is distributed under the terms of the Creative Commons Attribution 4.0 International License (http://creativecommons.org/licenses/by/4.0/)
