# Supplementary material for: Growth in ataxia telangiectasia
Source: Orphanet J Rare Dis. 2021 Mar 10;16:123. doi: 10.1186/s13023-021-01716-5 (PMC7945359; doi:10.1186/s13023-021-01716-5)
Supplement: Supplementary file 3 — Additional file 3. Dot plots showing raw data for growth charts. [file 13023_2021_1716_MOESM3_ESM.pdf]

Additional file 3

Dot plots showing raw data for growth charts

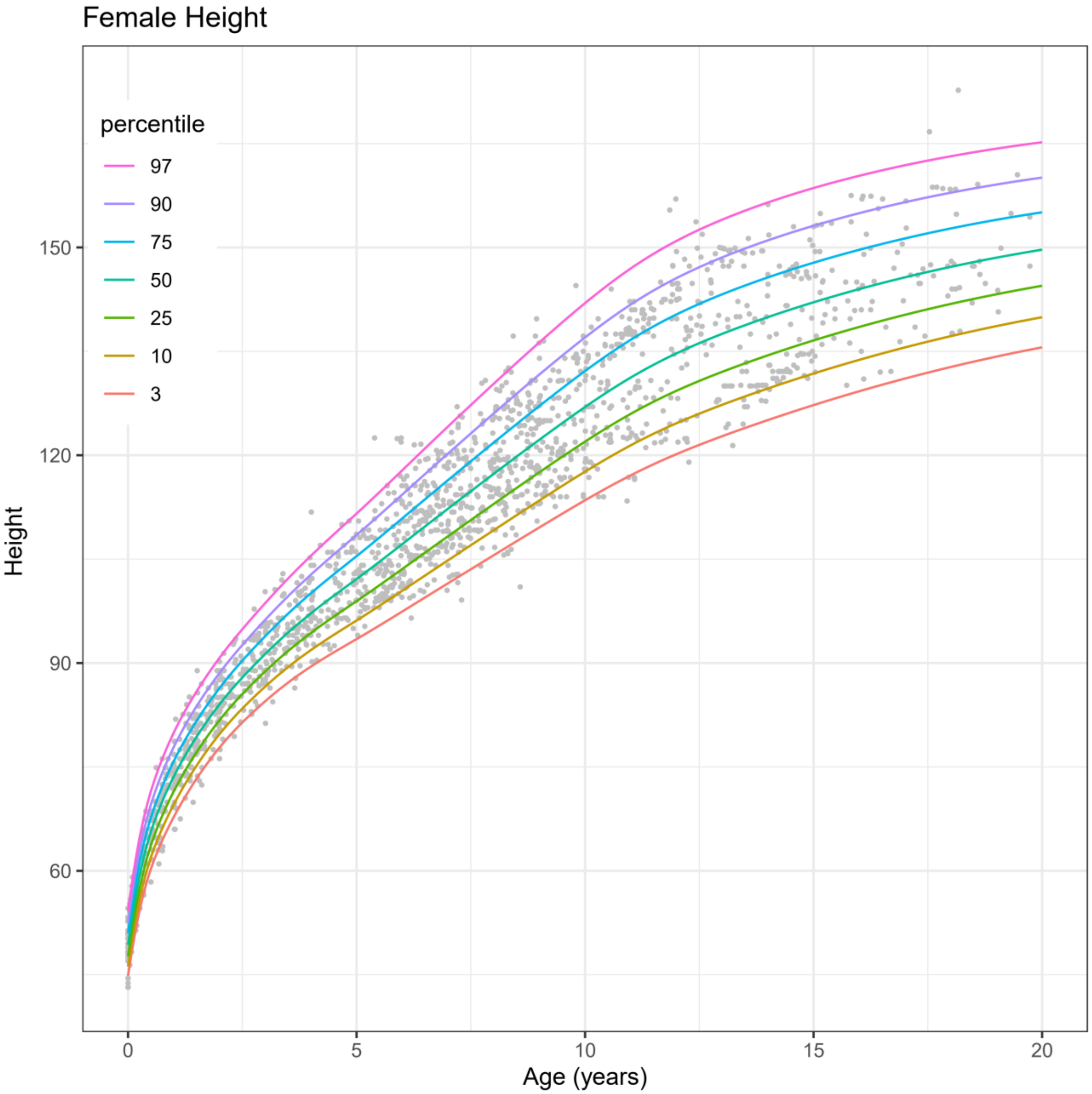

Additional Fig S11. Raw data for height in females with classic A-T.

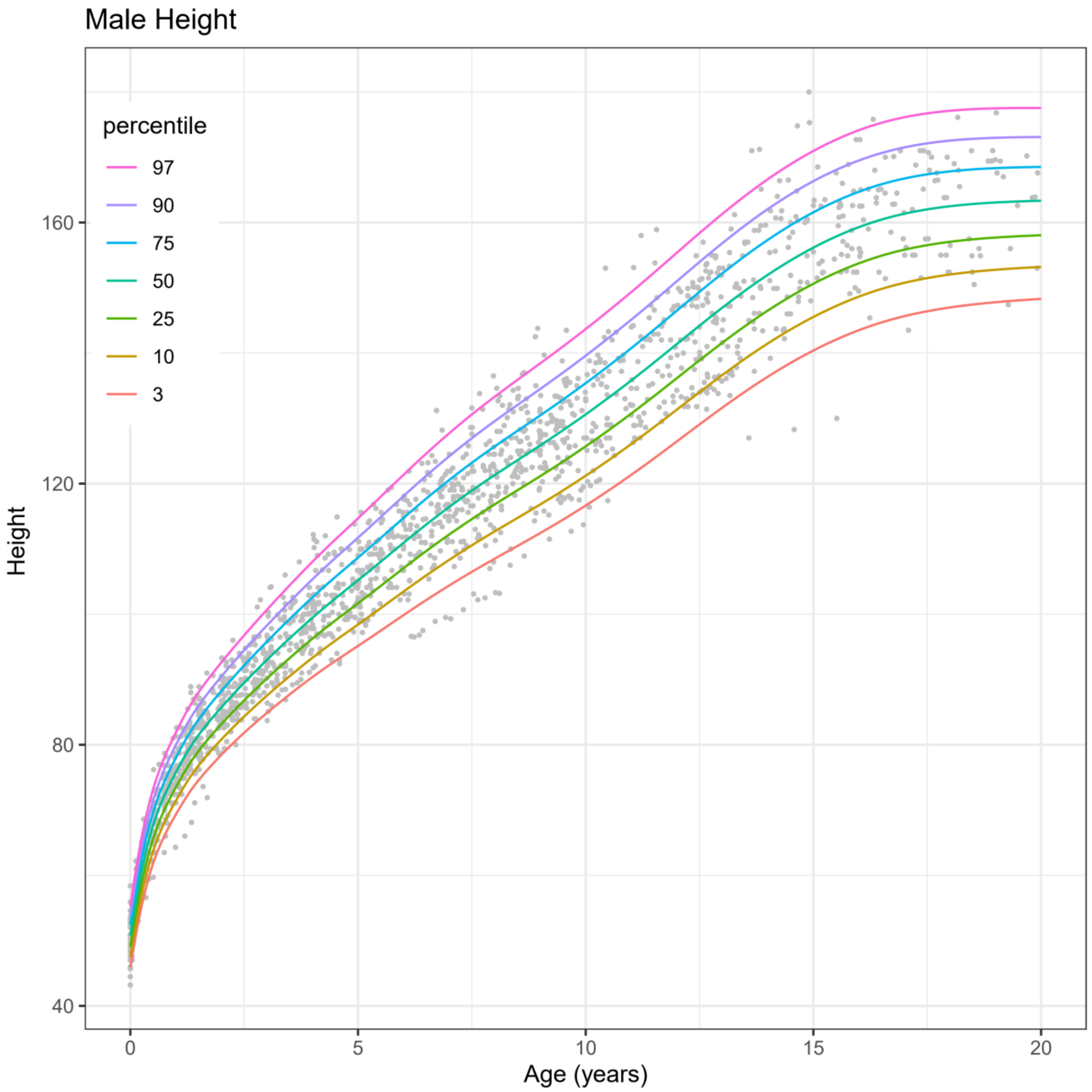

Additional Fig S12. Raw data for height in males with classic A-T.

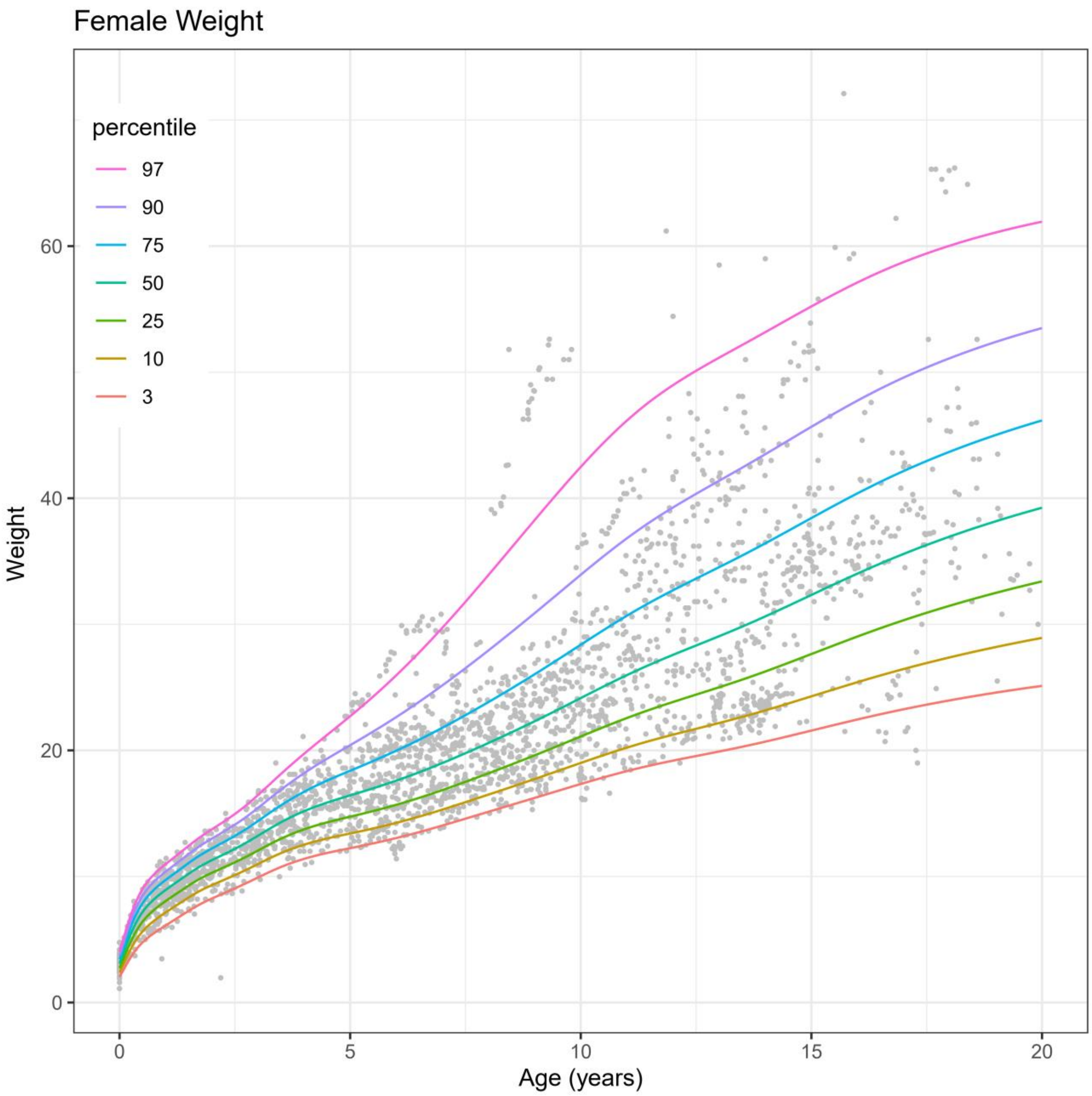

Additional Fig S13. Raw data for weight in females with classic A-T.

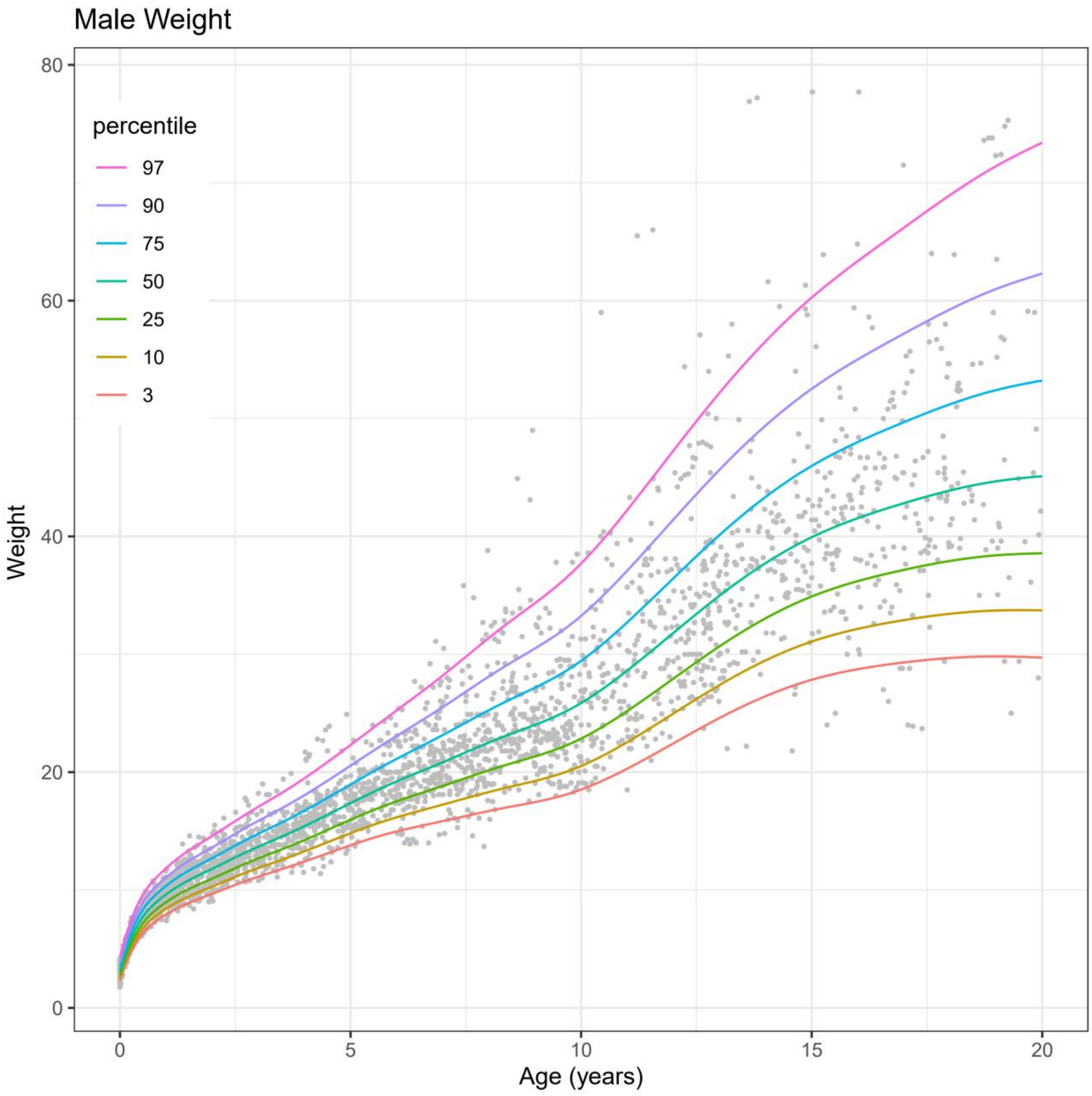

Additional Fig S14. Raw data for weight in males with classic A-T.

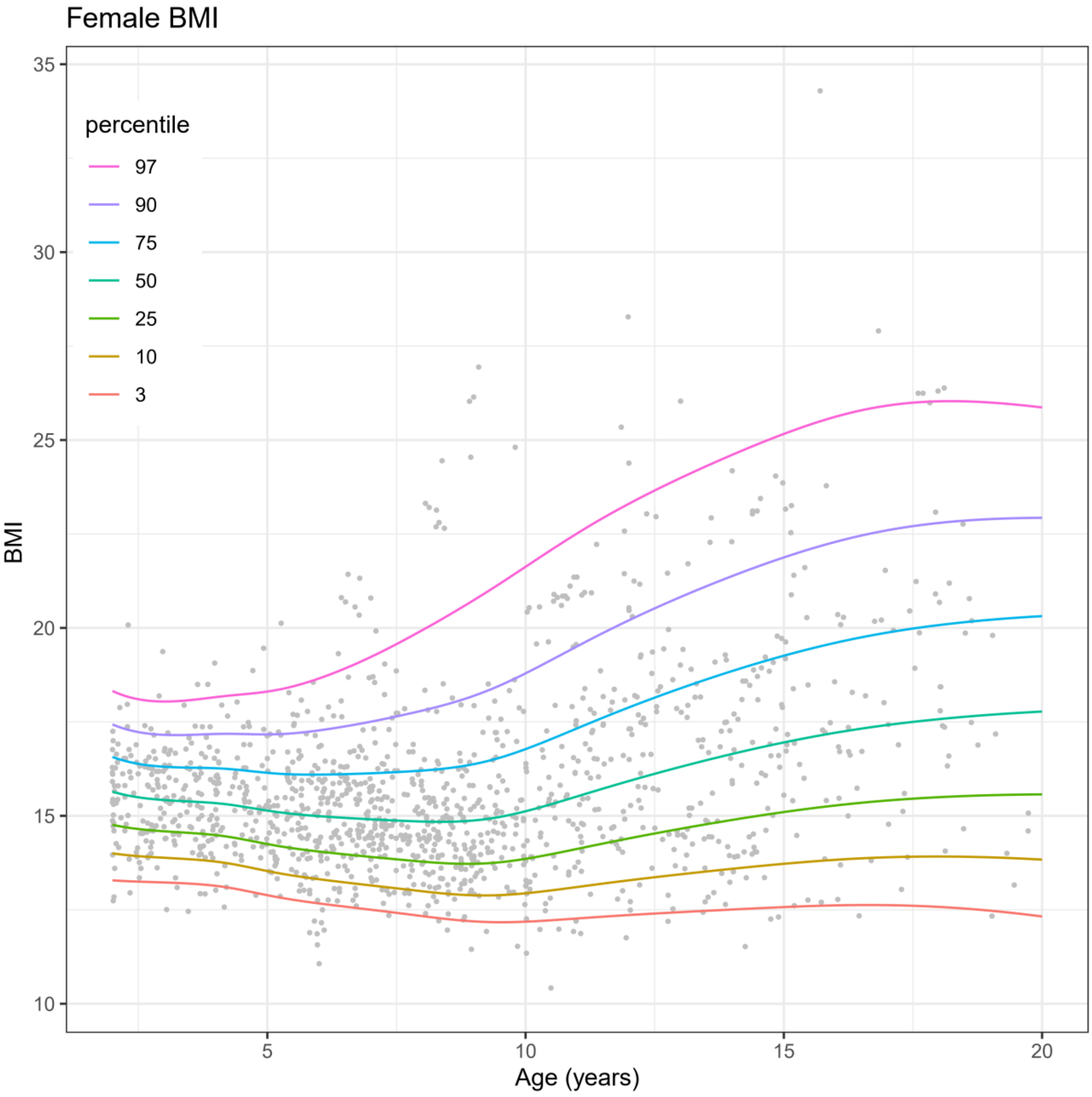

Additional Fig S15. Raw data for BMI in females with classic A-T.

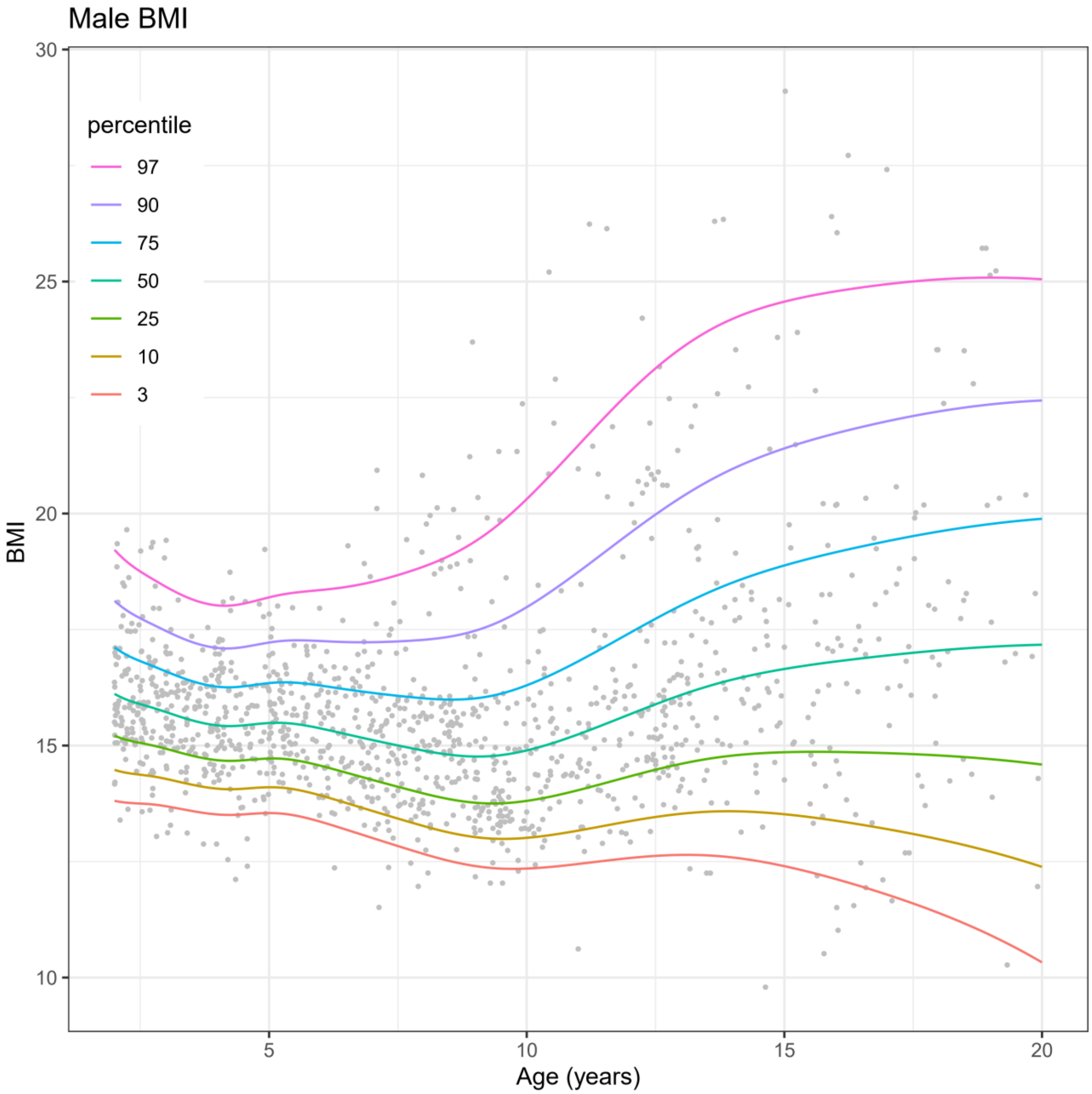

Additional Fig S16. Raw data for BMI in females with classic A-T.
