# Supplementary material for: Growth in ataxia telangiectasia
Source: Orphanet J Rare Dis. 2021 Mar 10;16:123. doi: 10.1186/s13023-021-01716-5 (PMC7945359; doi:10.1186/s13023-021-01716-5)
Supplement: Supplementary file 4 — Additional file 4. Additional figures S17–S21. [file 13023_2021_1716_MOESM4_ESM.pdf]

# Additional file 4

# Additional figures S17 – S21

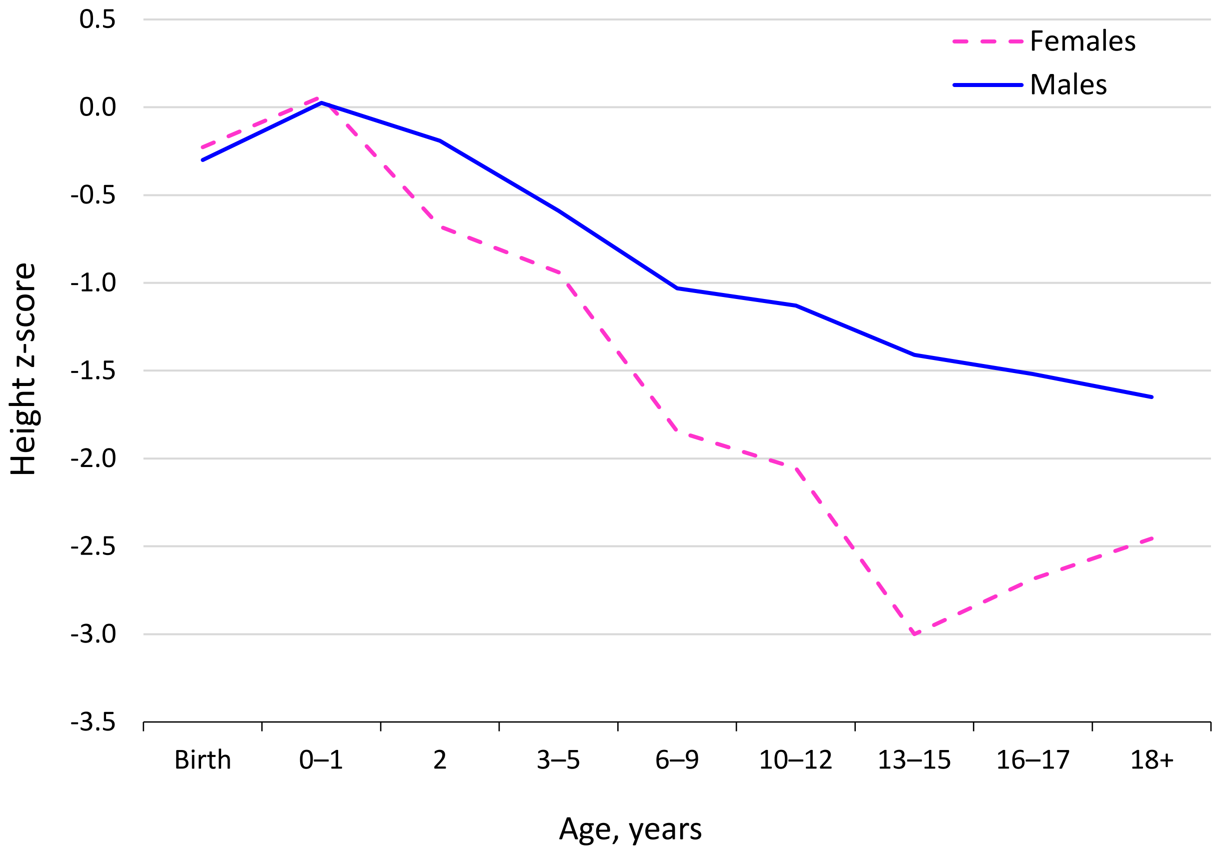


**Additional Figure S17.** Height z-score (relative to CDC) by age and sex in classic A-T patients. Height faltered in both sexes but more in females, reaching a minimum at age 14-15. The figure uses data from the extracted dataset (81 males, 81 females).

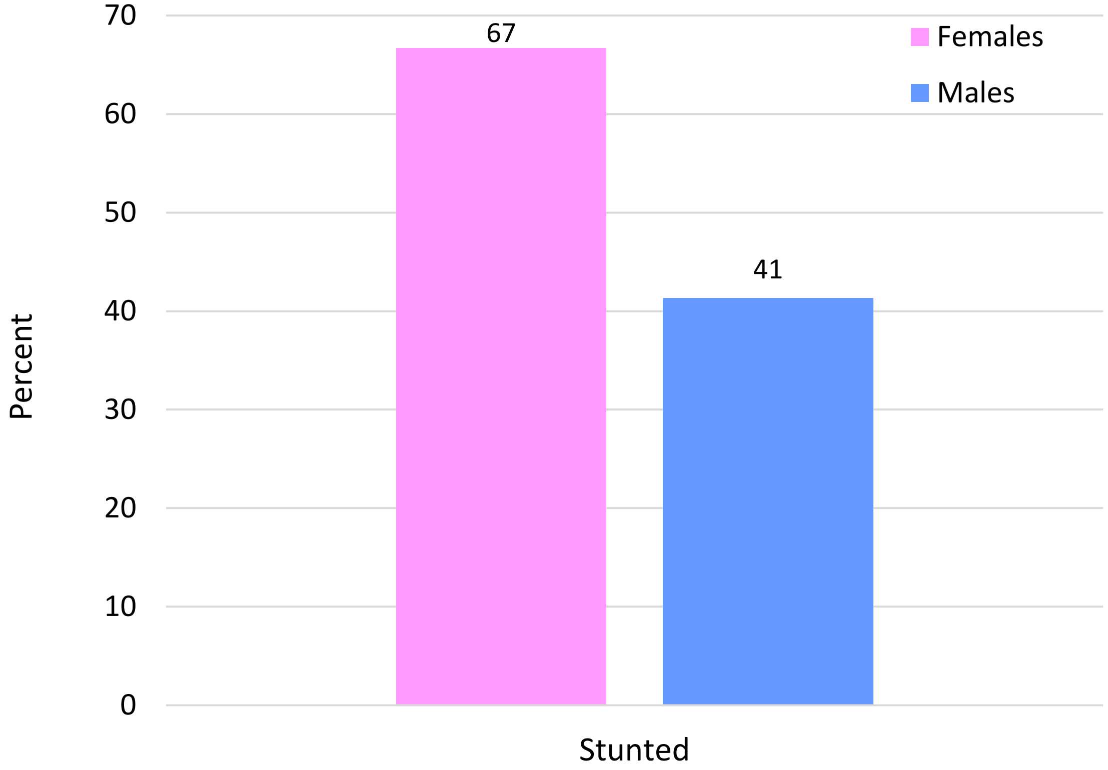


**Additional figure S18.** Percentage of stunted classic A-T patients by sex. Stunting is defined as a height z-score < –2. Stunting was more common in females. This figure is based on data from 88 male and female patients aged 13+ ($p = 0.02$).

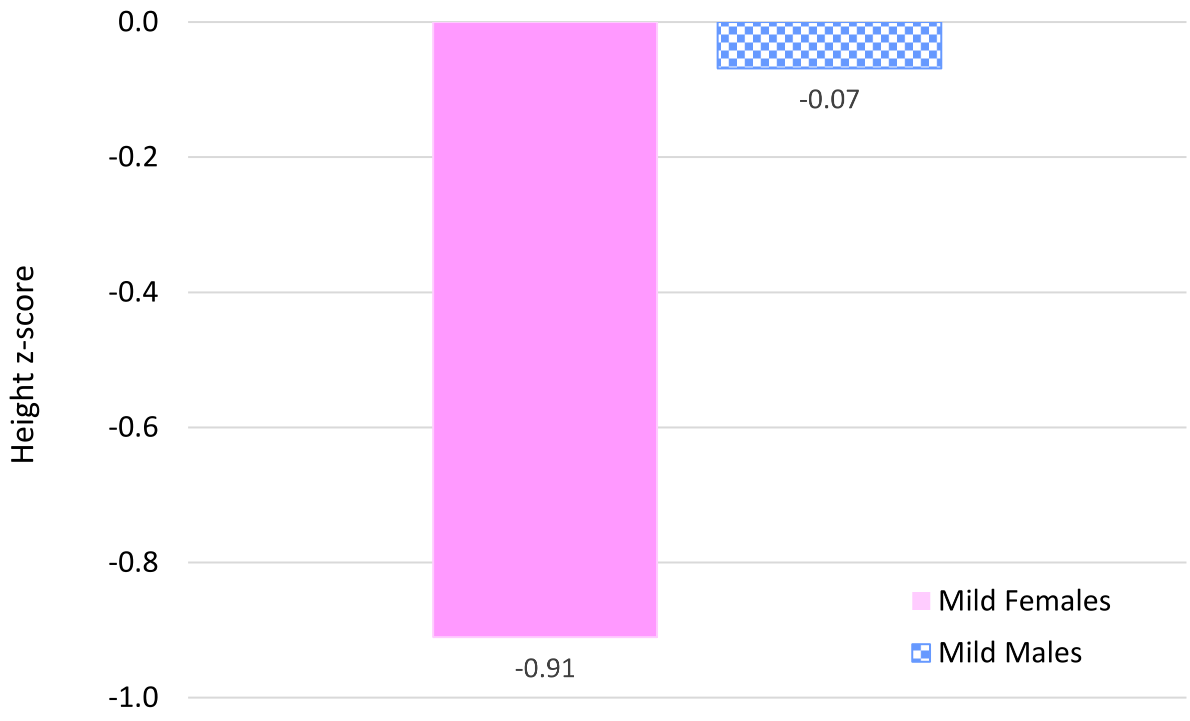


**Additional figure S19.** There is a sex-specific difference in height in mild A-T. Mean height z-score by sex in mild A-T patients aged ≥ 15 years (compared to CDC data). Data from 12 females and 9 males aged 15+ ($p<0.01$).

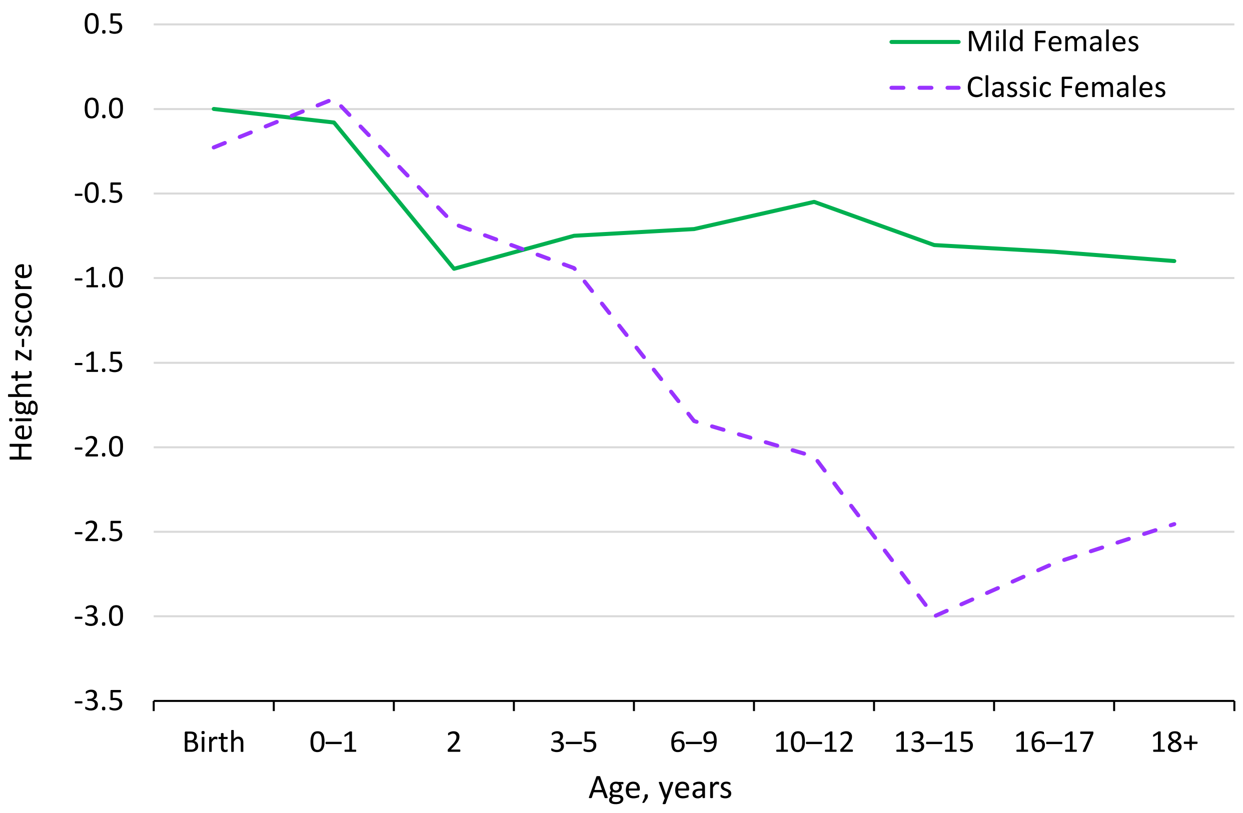


**Additional figure S20**. Median height z-score by age in females with classic and mild A-T. We did not have enough data to make a figure for males. The figure uses all data from the 81 females in the extracted dataset and data the 16 females with mild A-T.

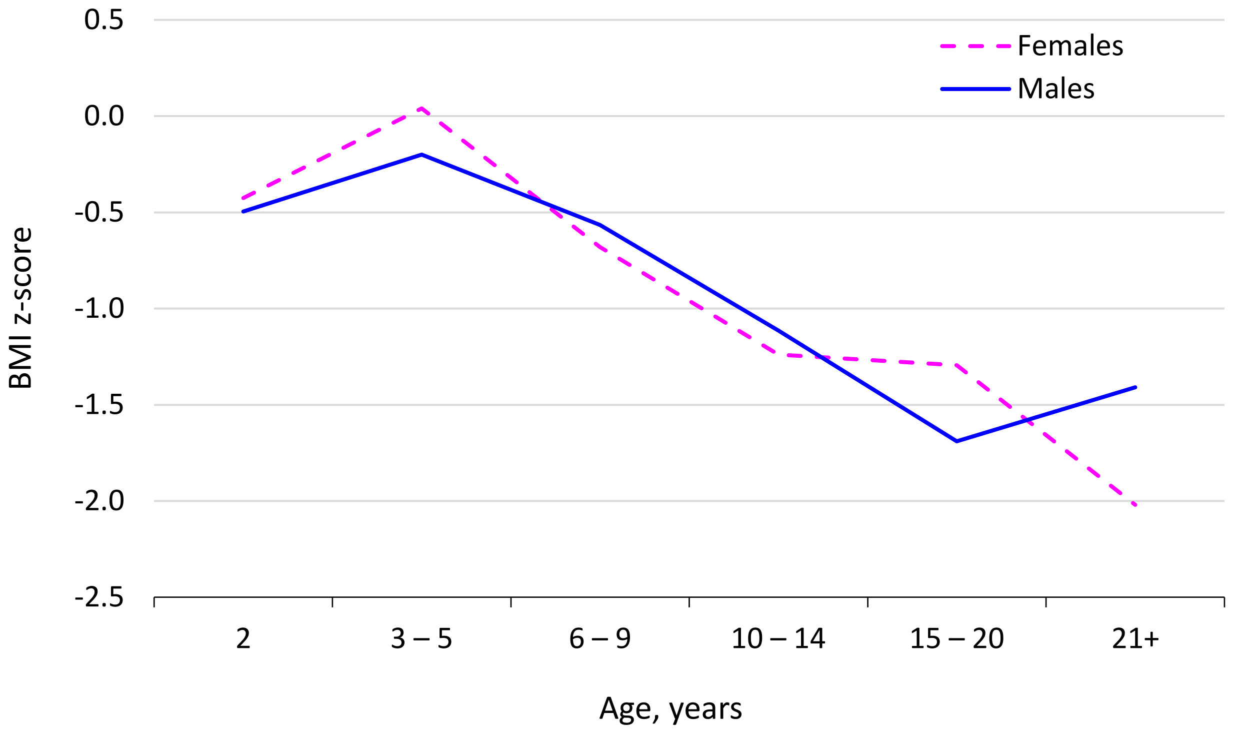


**Additional figure S21.** BMI z-score by age in classic A-T. BMI in classic A-T falls with age. The figure uses data from the extracted dataset (81 males, 81 females).
